# Hydroxylation site–specific and production-dependent effects of endogenous oxysterols on cholesterol homeostasis: Implications for SREBP-2 and LXR

**DOI:** 10.1016/j.jbc.2022.102733

**Published:** 2022-11-22

**Authors:** Hodaka Saito, Wakana Tachiura, Mizuki Nishimura, Makoto Shimizu, Ryuichiro Sato, Yoshio Yamauchi

**Affiliations:** 1 Laboratory of Food Biochemistry, Department of Applied Biological Chemistry, Graduate School of Agricultural and Life Sciences, The University of Tokyo, Tokyo, Japan; 2 Nutri-Life Science Laboratory, Department of Applied Biological Chemistry, Graduate School of Agricultural and Life Sciences, The University of Tokyo, Tokyo, Japan; 3 AMED-CREST, Japan Agency for Medical Research and Development, Tokyo, Japan

**Keywords:** cholesterol, cholesterol metabolism, cholesterol regulation, lipid metabolism, lipid signaling, oxysterols, SREBP-2, LXR, CH25H, CYP27A1, CYP46A1, ABCA1, ABC transporter A1, ABCG1, ABC transporter G1, ATF4, activating transcription factor 4, cDNA, complementary DNA, CHO, Chinese hamster ovary cell, diHC, 7α,25-dihydroxycholesterol, DMEM, Dulbecco's modified Eagle's medium, Dox, doxycycline, 24,25-EC, 24,25-epoxycholesterol, ER, endoplasmic reticulum, FBS, fetal bovine serum, HC, hydroxycholesterol, 25-HC, 25-hydroxycholesterol, 27-HC, 27-hydroxycholesterol, 7α-HC, 7α-hydroxycholesterol, HEK293T, human embryonic kidney 293T cell line, HMGCR, 3-hydroxy-3-methylglutaryl-CoA reducatase, Insig, insulin-induced gene, KDO, Kdo2-Lipid A, LPS, lipopolysaccharide, LXR, liver X receptor, PM, plasma membrane, PRH, primary rat hepatocyte, RXR, retinoid X receptor, SCAP, SREBP cleavage–activating protein, 24S-HC, 24S-hydroxycholesterol, SREBP-2, sterol regulatory element–binding protein-2, TLR4, Toll-like receptor 4

## Abstract

The cholesterol metabolites, oxysterols, play central roles in cholesterol feedback control. They modulate the activity of two master transcription factors that control cholesterol homeostatic responses, sterol regulatory element–binding protein-2 (SREBP-2) and liver X receptor (LXR). Although the role of exogenous oxysterols in regulating these transcription factors has been well established, whether endogenously synthesized oxysterols similarly control both SREBP-2 and LXR remains poorly explored. Here, we carefully validate the role of oxysterols enzymatically synthesized within cells in cholesterol homeostatic responses. We first show that SREBP-2 responds more sensitively to exogenous oxysterols than LXR in Chinese hamster ovary cells and rat primary hepatocytes. We then show that 25-hydroxycholesterol (25-HC), 27-hydroxycholesterol, and 24S-hydroxycholesterol endogenously synthesized by CH25H, CYP27A1, and CYP46A1, respectively, suppress SREBP-2 activity at different degrees by stabilizing Insig (insulin-induced gene) proteins, whereas 7α-hydroxycholesterol has little impact on SREBP-2. These results demonstrate the role of site-specific hydroxylation of endogenous oxysterols. In contrast, the expression of CH25H, CYP46A1, CYP27A1, or CYP7A1 fails to induce LXR target gene expression. We also show the 25-HC production–dependent suppression of SREBP-2 using a tetracycline-inducible CH25H expression system. To induce 25-HC production physiologically, murine macrophages are stimulated with a Toll-like receptor 4 ligand, and its effect on SREBP-2 and LXR is examined. The results also suggest that *de novo* synthesis of 25-HC preferentially regulates SREBP-2 activity. Finally, we quantitatively determine the specificity of the four cholesterol hydroxylases in living cells. Based on our current findings, we conclude that endogenous side-chain oxysterols primarily regulate the activity of SREBP-2, not LXR.

Cholesterol homeostasis is strictly controlled by multiple feedback mechanisms (1, 2, 3), and its dysregulation is associated with diverse pathophysiological settings from congenital to acquired human diseases (4, 5, 6). Not only cholesterol but also other sterol molecules, including oxysterols, participate in the feedback controls. The cholesterol metabolites, oxysterols, serve as crucial mediators that regulate cellular cholesterol homeostasis (7). In particular, oxysterols whose side chain is hydroxylated (hereafter referred to as side-chain oxysterols) exert much more potent effects than sterol-ring oxysterols. The oxysterol-mediated control of cholesterol homeostasis involves two distinct transcription factors; sterol regulatory element–binding protein-2 (SREBP-2) and liver X receptor (LXR). SREBP-2 regulates the expression of most enzymes involved in cholesterol biosynthesis and low-density lipoprotein receptor that mediates cholesterol uptake from the extracellular milieu (8, 9). On the other hand, the nuclear receptor LXR regulates the removal of excess cellular cholesterol by enhancing the expression of several ABC transporters that export cholesterol from cells, including ABC transporter A1 (ABCA1) and ABC transporter G1 (ABCG1) (6, 10).

SREBP-2 is a membrane-bound transcription factor. As an inactive form, SREBP-2 resides in the endoplasmic reticulum (ER), where it forms a complex with SREBP cleavage–activating protein (SCAP) and Insig (insulin-induced gene) proteins (9). SCAP serves as an SREBP-2 chaperone for its transport to the Golgi, whereas Insig acts as an ER retention factor for SREBP-2 (1). For the activation, the SREBP-2–SCAP complex dissociates from Insig and is transported to the Golgi for the proteolytic cleavage and release of the transcriptionally active domain. Its transport to the Golgi is tightly regulated by cholesterol and oxysterols. Cholesterol binds to SCAP, which leads to its conformational change for blocking the incorporation of the SREBP-2–SCAP complex into the COP-II vesicles (11, 12), whereas oxysterols interact with Insig proteins and prevent their proteasomal degradation (13). These events cooperatively prevent the exit of SREBP-2 from the ER and thus negatively regulate cholesterol biosynthesis and uptake (1). Another oxysterol effector, LXR, exhibits high-affinity binding to various oxysterols (14). When added to the culture medium, oxysterols bind to LXR as its ligand and activate its transcriptional activity. LXR forms a heterodimer with another nuclear receptor, retinoid X receptor (RXR), and transactivates the target genes to promote cellular cholesterol efflux.

Cholesterol undergoes oxygenation through enzymatic and nonenzymatic processes, and a variety of sterol hydroxylases catalyze the synthesis of oxysterols (15, 16). Although a number of oxysterols are identified in the circulation and tissues, the major oxysterols found in the body are 7α-hydroxycholesterol (7α-HC), 27-hydroxycholesterol (27-HC), 24S-hydroxycholesterol (24S-HC), and 25-hydroxycholesterol (25-HC). These four oxysterols are largely synthesized by CYP7A1, CYP27A1, CYP46A1, and CH25H, respectively, in the ER or mitochondria (16, 17). 27-HC, 24S-HC, and 25-HC are all capable of regulating both SREBP-2 and LXR when added to cells exogenously. On the other hand, 7α-HC, a major bile acid precursor, elicits only subtle effects on these two effectors.

As such, it is well established that oxysterols have dual roles in cholesterol homeostasis: regulation of cholesterol acquisition (biosynthesis and uptake) and cellular cholesterol removal. However, there has been controversy regarding the role of oxysterols in cholesterol homeostasis (7, 18). One of the reasons is that the current knowledge on oxysterol-mediated regulation is primarily based on experimental utilization of supraphysiological levels of oxysterol added exogenously to cells (hereafter referred to as “exogenous” oxysterols), despite the evidence that oxysterols are present in the circulation and tissues at low levels (15, 19). Little is known on whether endogenously synthesized oxysterols (hereafter referred to as “endogenous” oxysterols) elicit the same effects as exogenous oxysterols on cellular cholesterol homeostasis. To address these issues, we seek to explore whether endogenous oxysterols also play dual roles in cellular cholesterol homeostasis like exogenous oxysterols by expressing the four major cholesterol hydroxylases CH25H, CYP27A1, CYP46A1, and CYP7A1 in wildtype and mutant Chinese hamster ovary (CHO) cells that have extensively been utilized for studying cholesterol homeostatic responses. To confirm the results with CHO cells, we employ primary rat hepatocytes (PRHs) and murine macrophages as more physiological cell models. Finally, we quantitatively determine the specificity of these hydroxylases in living cells.

## Results

### SREBP-2 is more sensitive to exogenous side-chain oxysterols than LXR

**Figure 1:**
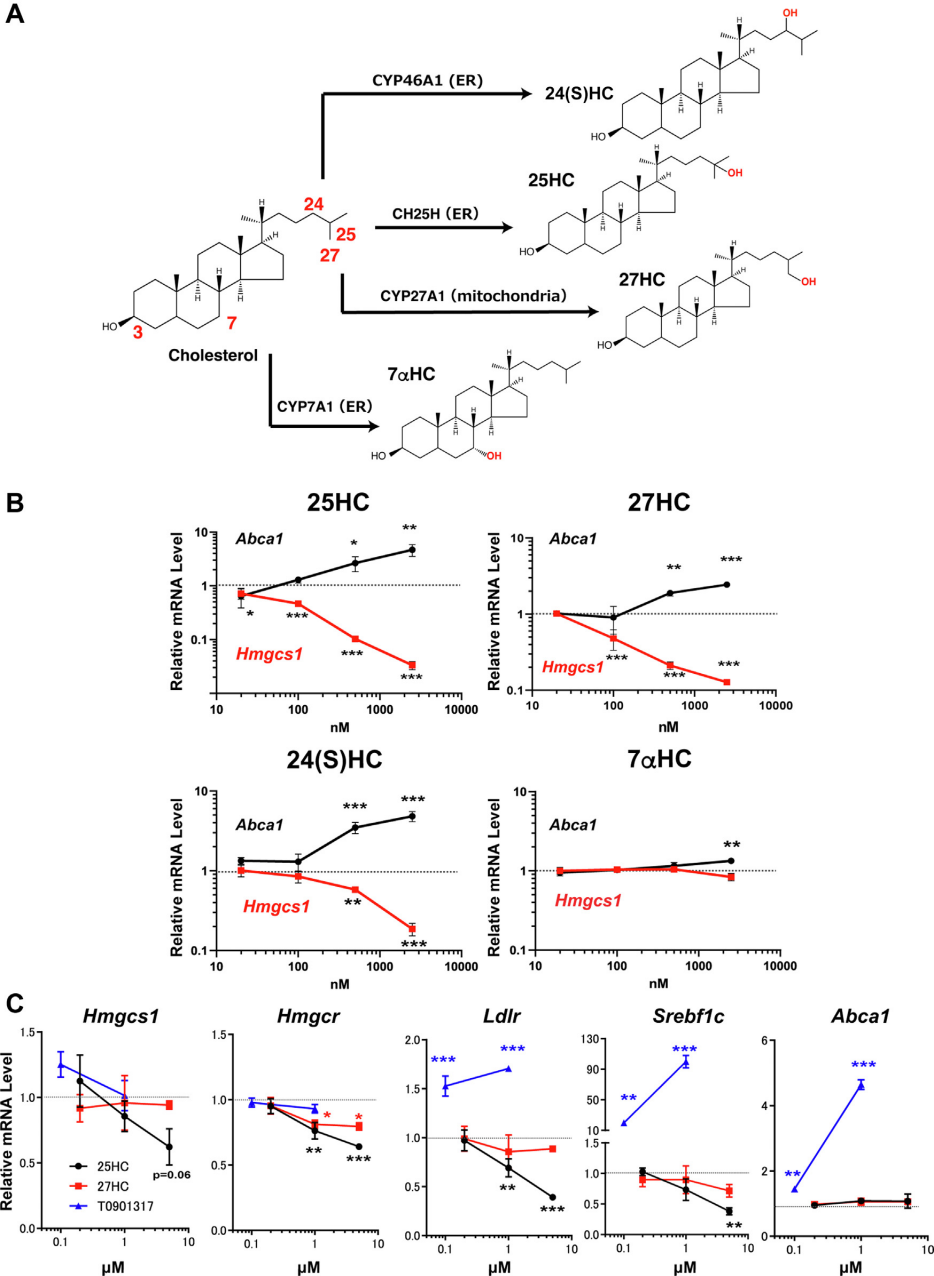
**SREBP-2 is more sensitive to exogenous side-chain oxysterols than LXR.***A*, biosynthesis of oxysterols by cholesterol hydroxylases, CH25H, CYP27A1, CYP46A1, and CYP7A1. *B*, CHO-K1 cells were treated without or with 25-HC, 27-HC, 24S-HC, or 7α-HC at different concentrations (0, 20, 100, 500, and 2500 nM) for 24 h. mRNA levels of SREBP-2 and LXR target genes (*Hmgcs1* and *Abca1*, respectively) were analyzed by qPCR. Data represent means ± SD (n = 3). Statistical analyses were performed by one-way ANOVA with Dunnett post hoc test by comparing to the vehicle treatment group (∗*p* < 0.05, ∗∗*p* < 0.01, and ∗∗∗*p* < 0.001). *C*, PRHs were treated with 25-HC, 27-HC, or T0901317 for 16 h at the indicated concentration. mRNA levels of SREBP-2 target genes (*Hmgcs1*, *Hmgcr*, and *Ldlr*) and LXR target genes (*Srebf1c* and *Abca1*) were determined by qPCR. Data represent means ± SD (n = 3). Statistical analyses were performed as aforementioned. CHO, Chinese hamster ovary cell; 7α-HC, 7α-hydroxycholesterol; 25-HC, 25-hydroxycholesterol; 27-HC, 27-hydroxycholesterol; 24S-HC, 24S-hydroxycholesterol; LXR, liver X receptor; PRH, primary rat hepatocyte; qPCR, quantitative PCR; SREBP-2, sterol regulatory element–binding protein-2.

We selected four major oxysterols enzymatically synthesized in the body, 7α-HC, 24S-HC, 25-HC, and 27-HC (Fig. 1*A*). Minimum amounts of each oxysterol required for modulating SREBP-2 and LXR activities are not fully characterized in a single-cell system. Therefore, we first carefully validated the dose-dependent effects of these four oxysterols on cholesterol homeostatic responses in CHO-K1 cells. To determine the dose dependence of oxysterols added exogenously, the cells were treated with 7α-HC, 24S-HC, 25-HC, or 27-HC at different concentrations. The results show that all the side-chain oxysterols suppress the expression of SREBP-2 target genes (*Hmgcs1*, *Hmgcr*, *Sqs*, and *Lss*) in a dose-dependent manner, but their inhibitory potencies are dependent on hydroxylation sites (Figs. 1*B* and S1*A*–*D*). In particular, 25-HC has the most potent inhibitory effect on the expression of the SREBP-2 target genes; it inhibits *Hmgcs1* expression at as low as 20 nM. 27-HC and 24S-HC also suppressed the expression of SREBP-2 target genes when added at 100 nM, but their inhibitory effects were lower than 25-HC. In contrast to the repression of SREBP-2 target genes at low concentrations, 25-HC, 27-HC, and 24S-HC failed to increase the expression of the LXR target genes (*Abca1* and *Abcg1*) at 100 nM or lower (Figs. 1*B* and S1*A*–*D*). Five hundred nanomolar or higher concentrations of oxysterols were required for upregulating the expression of LXR target genes. On the other hand, 7α-HC had no or negligible effects on mRNA levels of SREBP-2 and LXR target genes even at 2.5 μM. A control experiment showed that the synthetic LXR ligand T0901317 induces *Abca1* expression by fourfolds to fivefolds (Fig. S1*E*), indicating that CHO cells can respond to LXR ligands.

To confirm the results with CHO cells, we next conducted similar experiments using PRHs. The results show that 25-HC suppresses the expression of not only the SREBP-2 response genes but also the LXR response gene *Srebf1c* when PRHs are treated for 16 h (Fig. 1*C*). These results are in good agreement with previous reports showing that SREBP-2 activity is required for LXR-dependent SREBP-1c expression in rat hepatoma cells (20) and mouse livers (21). Shorter treatment (6 h) of PRHs with 25-HC inhibited the expression of *Hmgcs1* but not *Srebf1c* (Fig. S2), supporting this notion. Consistent with the results with CHO cells, 27-HC exhibited weaker effects on the suppression of SREBP-2 target gene expression than 25-HC. In addition, higher oxysterol concentrations were necessary to inhibit SREBP-2 target gene expression in PRHs than CHO cells, presumably because they express CYP7B1 that converts 25-HC and 27-HC to 7α,25-diHC and 7α,27-diHC, respectively. Both 25-HC and 27-HC did not upregulate *Abca1* expression either at 6 h or 16 h, suggesting that *Srebf1c* is a predominant LXR response gene in hepatocytes as the synthetic LXR ligand T0901917 much more robustly transactivates *Srebf1c* than *Abca1*. These results indicate that side-chain oxysterols preferentially downregulate SREBP-2 in both CHO cells and PRHs. At higher concentrations, they also activate LXR without adding RXR ligands in CHO cells but not in PRHs.

### Cholesterol hydroxylase expression modulates cholesterol homeostatic responses

**Figure 2:**
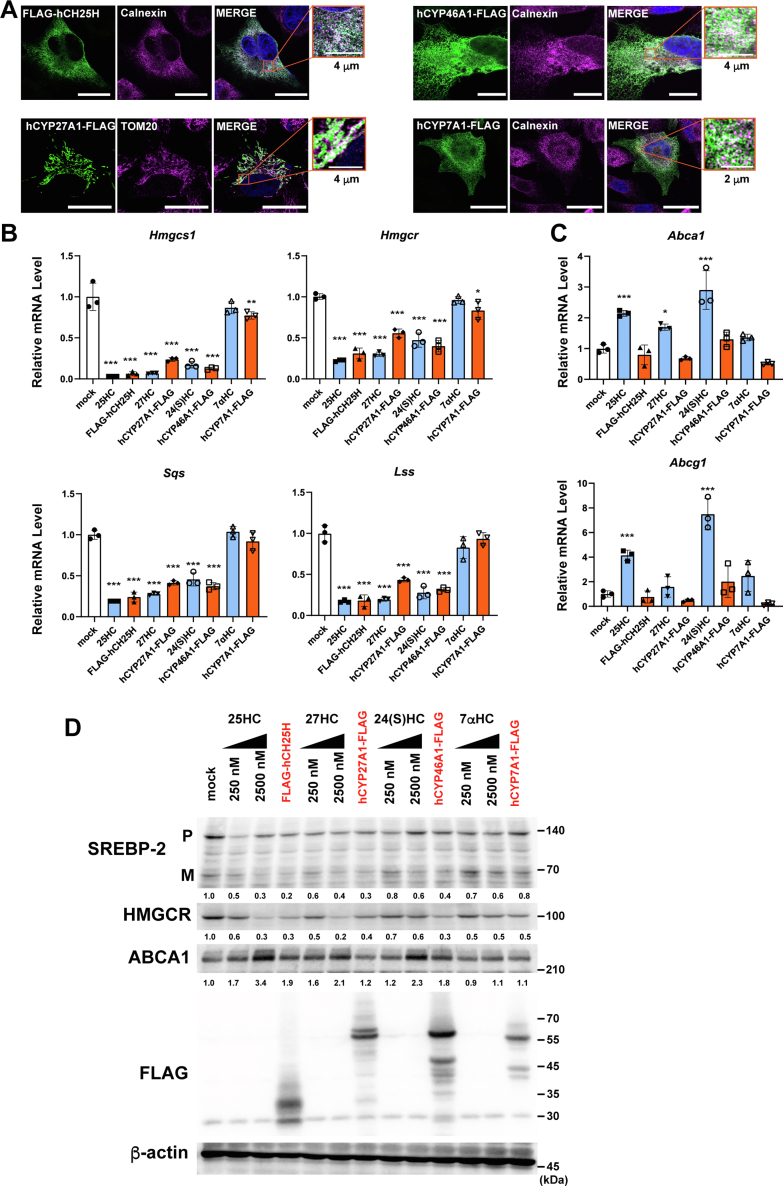
**Effect of cholesterol hydroxylase expression oncholesterol homeostatic responses.***A*, cellular localization of cholesterol hydroxylases. A2058 cells were transfected with the plasmids (pFLAG-CH25H, pCYP27A1-FLAG, pCYP46A1-FLAG, or pCYP7A1-FLAG) and fixed 2 days after transfection. Cholesterol hydroxylases were labeled with anti-FLAG antibody followed by Alexa 488-conjugated antimouse immunoglobulin G (IgG) (*green*). The endoplasmic reticulum (ER) and mitochondria were labeled with anticalnexin and anti-Tom20 antibodies, respectively, followed by Alexa 568-conjugated anti-rabbit IgG (*magenta*). Images were taken under a confocal microscopy. The scale bars (main fields) represent 20 μm; *insets* represent 2 or 4 μm as indicated. *B* and *C*, effect of hydroxylase expression on cholesterol homeostatic responses. CHO-K1 cells were transfected with plasmids as aforementioned and incubated for 48 h in medium containing 0.1% FBS. Cells transfected with pCMV10 (*mock*) were also treated without or with 2.5 μM oxysterols (25-HC, 27-HC, 24S-HC, or 7α-HC) for 16 h in medium containing 0.1% FBS before harvesting cells. mRNA levels of SREBP-2 target genes (*B*) and of LXR target genes (*C*) were analyzed by quantitative PCR. Data represent means ± SD (n = 3). Statistical analyses were performed by one-way ANOVA with Dunnett post hoc test by comparing to mock (∗*p* < 0.05, ∗∗*p* < 0.01, and ∗∗∗*p* < 0.001). *D*, effect of hydroxylase expression on SREBP-2 processing. CHO-K1 cells were transfected with the plasmids and treated as aforementioned. Before harvesting, cells were incubated with MG132 (20 μM) for 2 h. Expression of SREBP-2 (P, precursor; M, mature form), HMGCR, ABCA1, and FLAG-tagged hydroxylases were analyzed by immunoblot. β-actin serves as a loading control. Relative changes in SREBP-2 mature form, HMGCR, and ABCA1 from two experiments were indicated at the *bottom* of each blot. ABCA1, ABC transporter A1; CHO, Chinese hamster ovary cell; FBS, fetal bovine serum; 7α-HC, 7α-hydroxycholesterol; 25-HC, 25-hydroxycholesterol; 27-HC, 27-hydroxycholesterol; HMGCR, 3-hydroxy-3-methylglutaryl-CoA reducatase; 24S-HC, 24S-hydroxycholesterol.

Circulating oxysterol levels are as low as approximately 30 to 150 ng/ml (80–400 nM), which is 0.001 to 0.01% of circulating cholesterol (15). Although 25-HC, 27-HC, 24S-HC, and 7α-HC are all enzymatically synthesized from cholesterol within cells, the effects of cholesterol hydroxylase expression on cholesterol homeostatic responses remain poorly characterized at cellular levels. Therefore, we sought to explore how the expression of the major cholesterol hydroxylases, CH25H, CYP27A1, CYP46A1, or CYP7A1, alters cholesterol homeostatic responses in CHO-K1 cells. We first examined the cellular localization of CH25H, CYP27A1, CYP46A1, and CYP7A1 forcedly expressed. The results show that CH25H, CYP46A1, and CYP7A1 reside in the ER, whereas CYP27A1 localizes to the mitochondria, confirming their correct localization (Fig. 2*A*). Furthermore, CH25H, CYP46A1, and CYP7A1 partly colocalize with or closely localize to the outer mitochondrial membrane protein TOM20, suggesting the localization of these enzymes at the ER–mitochondria contact site known as mitochondria-associated membranes (Fig. S3). Next, we examined the effect of hydroxylase expression on SREBP-2 activity. The results show that the expression of CH25H, CYP27A1, and CYP46A1 markedly decreases mRNA levels of SREBP-2 target genes (*Hmgcs1*, *Hmgcr*, *Sqs*, and *Lss*), whereas CYP7A1 expression shows only subtle effects on the expression of these genes (Figs. 2*B* and S4*A*). Consistent with these results, the expression of CH25H, CYP27A1, and CYP46A1 substantially reduced the *Hmgcs1* promoter activity as much as the addition of the corresponding oxysterols (Fig. S4*B*). CYP7A1 expression slightly reduced the SREBP-2 activity, which is consistent with previous observations that 7α-HC inhibited SREBP-2 processing at higher concentrations (13). These results also suggest that the expression of these hydroxylases produces significant amounts of corresponding oxysterols. To confirm whether the effect of CH25H on SREBP-2 regulation depends on its hydroxylase activity, mutant CH25H (CH25H^H242Q/H243Q^), where two histidines (H242 and H243) in one of the three histidine boxes essential for its activity (22) are replaced with glutamines, was expressed in CHO cells. The results show that CH25H-dependent suppression of SREBP-2 response gene expression became sluggish in cells expressing CH25H^H242Q/H243Q^, indicating that the hydroxylase activity is required for SREBP-2 regulation (Fig. S4*C*). Furthermore, it was shown that in human embryonic kidney 293T (HEK293T) cells where the mitochondrial cholesterol transporter StAR (also known as STARD1) is lacking, StAR was required for suppressing mRNA levels of SREBP-2 target genes by CYP27A1 expression (Fig. S4*D*), confirming a previous report (23). We also monitored the expression of LXR target genes in cells overexpressing these four hydroxylases. In contrast to exogenous oxysterols at 2.5 μM enough high for induction, the expression of hydroxylases failed to increase mRNA levels of *Abca1* and *Abcg1* (Fig. 2*C*).

To further explore the effects of the hydroxylase expression on cholesterol homeostatic responses, we assessed SREBP-2 processing and 3-hydroxy-3-methylglutaryl-CoA reducatase (HMGCR) and ABCA1 protein expression. As expected, the expression of CH25H, CYP27A1, and CYP46A1, but not CYP7A1, markedly inhibited SREBP-2 processing as strongly as exogenous side-chain oxysterols (2.5 μM) did (Fig. 2*D*). Consistently, HMGCR protein expression was also substantially reduced by CH25H, CYP27A1, and CYP46A1. In contrast to the SREBP-2 pathway, expressing these hydroxylases had only modest effects on ABCA1 protein levels, whereas its expression was markedly induced by exogenous 25-HC, 27-HC, and 24S-HC. These results show that *de novo* synthesized side-chain oxysterols primarily regulate SREBP-2 activity but have little impact on LXR activity.

### 25-HC production–dependent inactivation of SREBP-2

**Figure 3:**
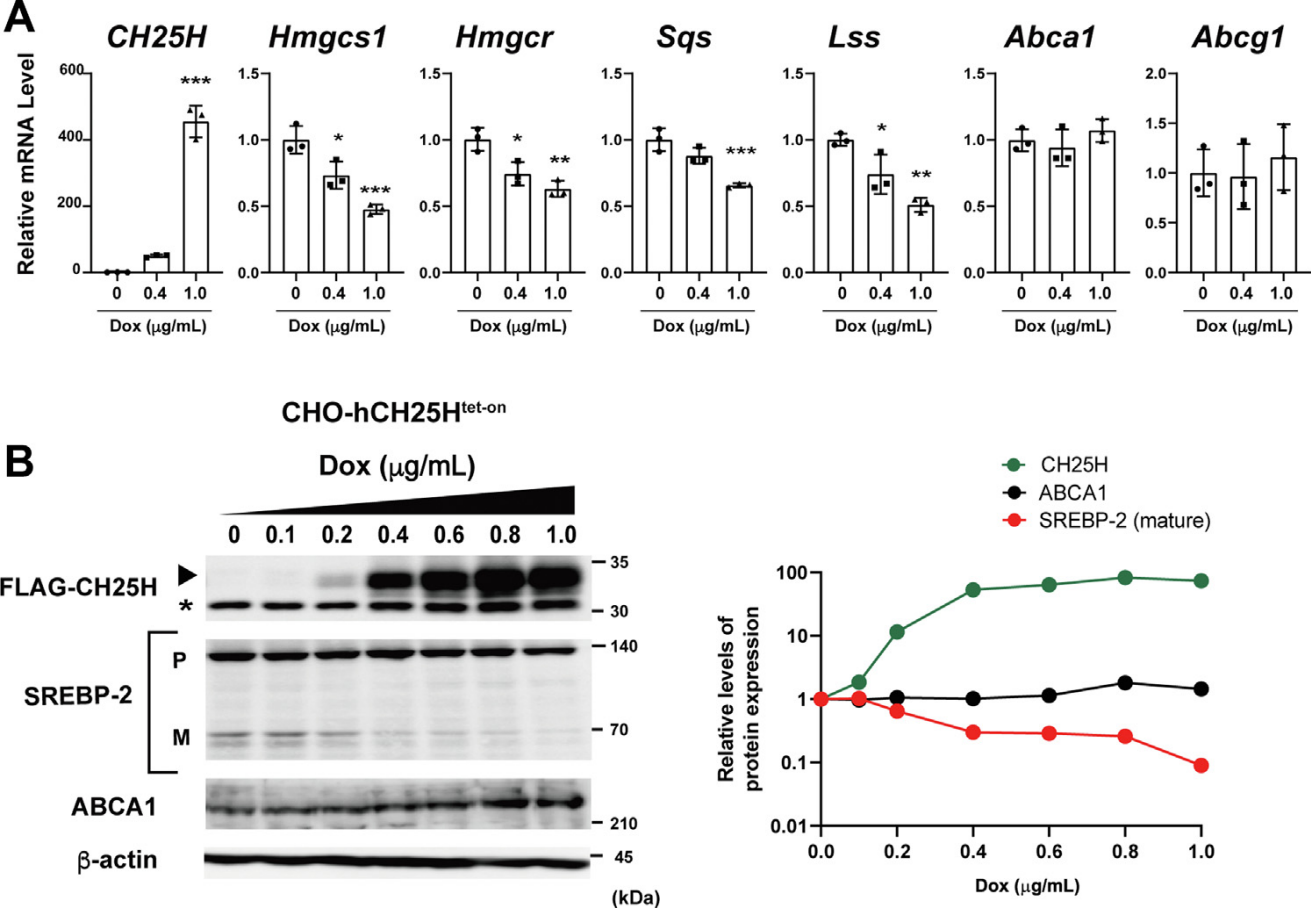
**CH25H expression level–dependent inactivation of SREBP-2.***A*, correlation between CH25H expression and SREBP-2 activity. CHO-CH25H^tet-on^ cells were treated without or with 0.4 and 1 μg/ml Dox for 24 h. mRNA levels of the indicated genes were measured by qPCR. Error bars represent SD from three biological replicates. Statistical analyses were performed by one-way ANOVA with Dunnett post hoc test (∗*p* < 0.05, ∗∗*p* < 0.01, and ∗∗∗*p* < 0.001). *B*, effect of CH25H expression levels on SREBP-2 processing and ABCA1 expression. CHO-CH25H^tet-on^ cells were incubated with different concentrations of Dox (0–1 μg/ml) for 24 h as indicated. Cell lysate was subjected to immunoblot analysis to detect FLAG-CH25H, SREBP-2 (both precursor [P] and mature [M] forms), and ABCA1. β-actin was detected as a loading control. The *asterisk* denotes a nonspecific band. In the *right panel*, relative changes in the expression of FLAG-CH25H, SREBP-2 mature form, and ABCA1 are plotted. ABCA1, ABC transporter A1; CHO, Chinese hamster ovary cell; Dox, doxycycline; qPCR, quantitative PCR; SREBP-2, sterol regulatory element–binding protein-2.

Our results described previously show that CH25H expression and its product 25-HC have a more potent inhibitory effect on SREBP-2 activity than other hydroxylases and oxysterols. To more carefully examine the impact of CH25H expression, we took advantage of the doxycycline (Dox)-inducible CH25H expression system that enables us to control the levels and timing of its expression. We established CHO-CH25H^tet-on^ cells and examined the effects of CH25H expression on cholesterol homeostatic responses. As expected, Dox induced CH25H expression in a manner dependent on its concentration (Fig. 3, *A* and *B*). We first examined the effects of increasing CH25H expression on cholesterol homeostatic responses at mRNA levels. The results show that the expression of SREBP-2 target genes (*Hmgcs1*, *Hmgcr*, *Sqs*, and *Lss*) is suppressed in a manner dependent on *CH25H* expression (Fig. 3*A*), but mRNA levels of LXR target genes (*Abca1* and *Abcg1*) are not altered by the induction of *CH25H* expression.

To directly demonstrate the CH25H-dependent repression of SREBP-2, we next examined SREBP-2 processing with an increasing concentration of Dox. We found the inverse correlation between CH25H expression and SREBP-2 processing (Fig. 3*B*); the magnitude of reduction in the mature form depended on CH25H expression levels. On the other hand, the increase in ABCA1 protein levels was only subtle even when the cells were treated with Dox at the concentration of 0.8 μg/ml or higher. These data are consistent with the results in Figure 2.

### Inactivation of SREBP-2 by physiological CH25H induction

**Figure 4:**
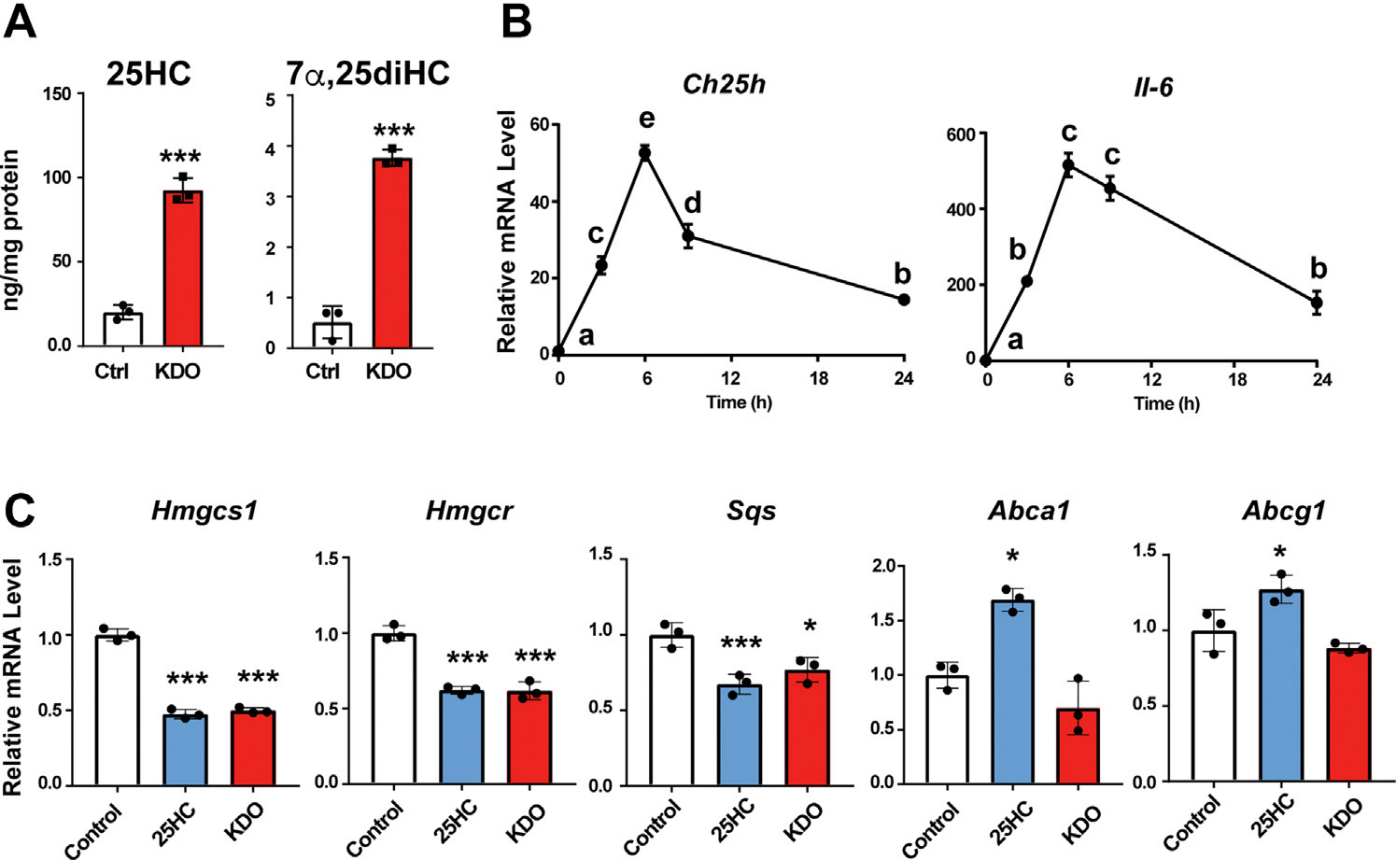
**Effect of *Ch25h* induction by a TLR4 ligand on SREBP-2 and LXR.***A*, TLR4 stimulation increases cellular contents of 25-HC in murine macrophages. J774 macrophages were incubated with or without KDO (100 ng/ml) for 20 h. Cellular 25-HC and 7α,25-diHC contents were determined by GC–MS/MS. Statistical analyses were performed by Student’s *t* test (∗∗∗*p* < 0.001). *B*, upregulation of *Ch25h* expression by TLR4 ligand. J774 macrophages were stimulated with KDO for 3, 6, 9, and 24 h. mRNA levels of *Ch25h* and *Il6* were measured. Data represent means ± SD (n = 3). Statistical analyses were performed by one-way ANOVA with Tukey–Kramer post hoc test. Different letters denote statistical significance (*p* < 0.05). *C*, inhibition of SREBP-2 response gene expression by TLR4 ligand. J774 macrophages were treated without or with 25-HC (2.5 μM) and KDO (100 ng/ml) for 9 h. mRNA levels of the indicated genes were determined. Data represent means ± SD (n = 3). Statistical analyses were performed by one-way ANOVA with Dunnett post hoc test (∗*p* < 0.05, ∗∗*p* < 0.01, and ∗∗∗*p* < 0.001). 7α,25-diHC, 7α,25-dihydroxycholesterol; 25-HC, 25-hydroxycholesterol; KDO, Kdo2-Lipid A; LXR, liver X receptor; SREBP-2, sterol regulatory element–binding protein-2; TLR4, Toll-like receptor 4.

CH25H expression is markedly induced by lipopolysaccharide (LPS), a Toll-like receptor 4 (TLR4) ligand, in macrophages (24). To test whether physiological levels of CH25H induction also inhibit SREBP-2 activity, we treated murine J774 macrophages with Kdo2-Lipid A (KDO), a selective TLR4 ligand, and examined the effect on SREBP-2 and LXR. As expected, KDO markedly increased cellular 25-HC and 7α,25-dihydroxycholesterol (diHC) contents (Fig. 4*A*). It robustly upregulated *Ch25h* mRNA expression in addition to the inflammatory gene Il6, and their expression declined 24 h after stimulation (Fig. 4*B*). We next assessed mRNA levels of SREBP-2 and LXR target genes in J774 macrophages treated with KDO or 25-HC for 9 h since KDO-dependent 25-HC production reached maximal levels 8 to 12 h after the stimulation (24). The results show that TLR4 stimulation substantially decreases mRNA levels of *Hmgcs1*, *Hmgcr*, and *Sqs* as much as exogenous 25-HC does (Fig. 4*C*). However, in contrast to exogenous 25-HC, KDO did not upregulate *Abca1* and *Abcg1* expression. These results support the aforementioned findings with CHO cells that endogenous 25-HC preferentially regulates SREBP-2.

### Endogenous side-chain oxysterols regulate Insig-dependent events

**Figure 5:**
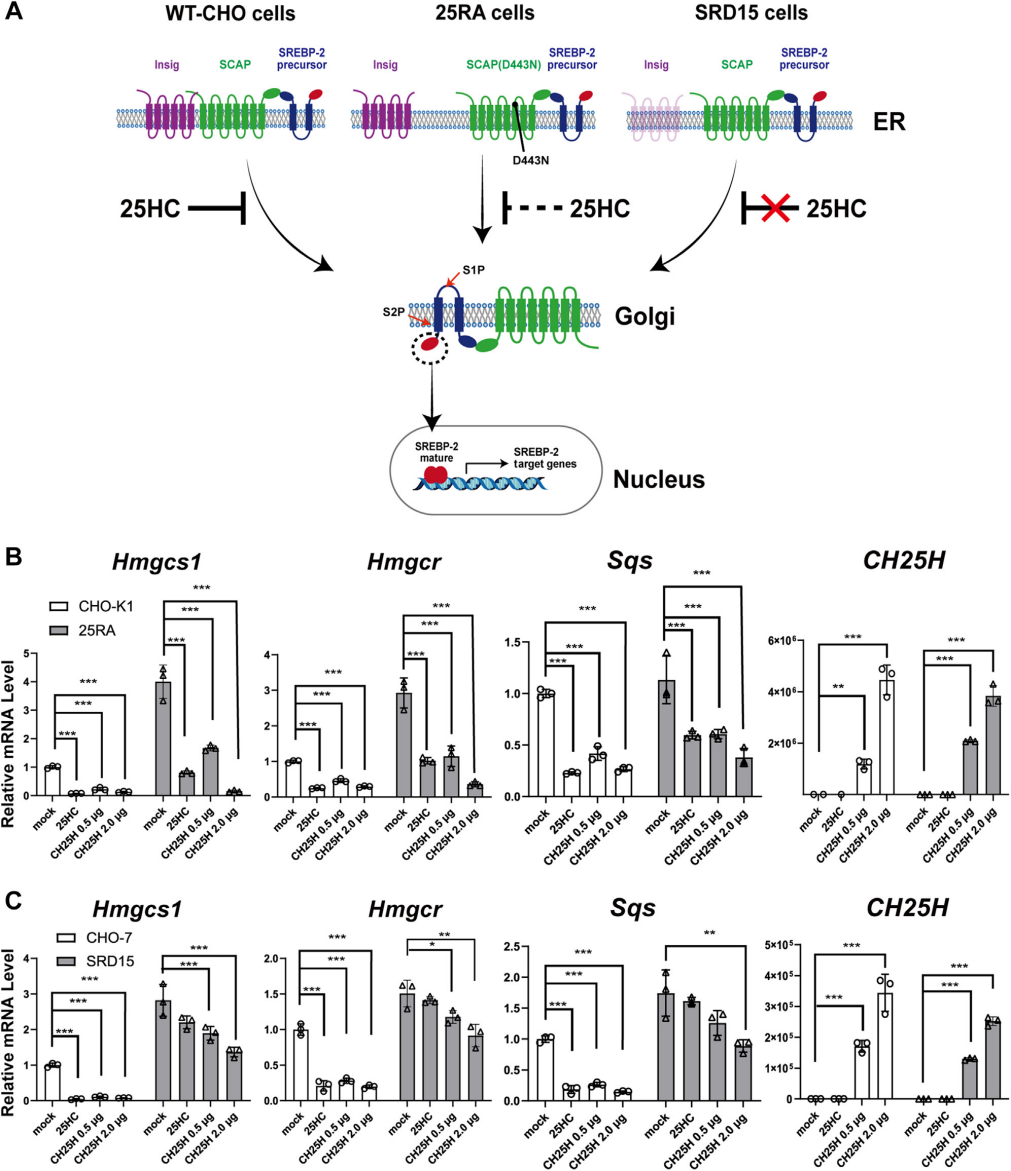
**Insigs are required forinactivation of SREBP-2 activity by endogenous oxysterols.***A*, diagram of constitutive activation of SREBP-2 in 25RA and SRD15 cells. In these two mutants, SREBP-2 is constitutively activated by different mechanisms. See text for more detail. *B* and *C*, expression of SREBP-2 target genes in 25RA (*B*) and SRD-15 (*C*) cells. On day 0, 25RA, SRD-15, and their parental cells (CHO-K1 and CHO-7, respectively) were seeded into 6-well plates. On day 1, cells were transfected with different amounts (0.5 and 2.0 μg/well) of plasmid to express FLAG-CH25H or an empty plasmid as indicated. On day 2, mock-transfected cells were either treated or untreated with 25-HC cells (2.5 μM) for 16 h. On day 3, cells were harvested for RNA isolation. mRNA levels of SREBP-2 target genes were analyzed by qPCR. Data represent means ± SD (n = 3). Statistical analyses were performed by one-way ANOVA with Dunnett post hoc test (∗*p* < 0.05, ∗∗*p* < 0.01, and ∗∗∗*p* < 0.001). CHO, Chinese hamster ovary; 25-HC, 25-hydroxycholesterol; Insig, insulin-induced gene; qPCR, quantitative PCR; SREBP-2, sterol regulatory element–binding protein-2.

The results in Figure 2, Figure 3, Figure 4 suggest that endogenous side-chain oxysterols selectively regulate SREBP-2 activity. SREBP-2 processing is regulated by SCAP and Insig at the ER by independent mechanisms; cholesterol binds and regulates SCAP, whereas side-chain oxysterols stabilize Insig by direct interaction. Next, we asked how endogenous oxysterols modulate SREBP-2 activity. To this end, we employed two CHO mutants, 25RA and SRD15, both of which exhibit 25-HC-resistant and constitutive SREBP-2 activation phenotypes through distinct mechanisms (Fig. 5*A*); 25RA cells express constitutively active mutant SCAP, SCAP^D443N^ (25), that renders SCAP insensitive to cholesterol (26), whereas SRD15 cells lack Insig-1 and Insig-2, negative regulators of SREBP (27). We transiently expressed CH25H in these mutant CHO cells and their parental cells and compared the differences in cholesterol homeostatic responses between mutant and parental cells. As previously reported (25, 27), in both 25RA and SRD15 cells, mRNA levels of most SREBP-2 target genes were approximately twofolds to threefolds of their parental cells (Fig. 5, *B* and *C*). The expression of CH25H in 25RA cells resulted in a marked reduction in mRNA levels of SREBP-2 target genes in a dose-dependent manner (Figs. 5*B* and S5*A*). However, in 25RA cells forcedly expressed CH25H or treated with 25-HC, mRNA levels of these genes were still higher than parental CHO-K1 cells with the same treatment, thereby providing 25RA cells with 25-HC resistance (25, 27). In sharp contrast to 25RA cells, the reduction in the expression of SREBP-2 target genes upon CH25H expression was much more modest in SRD15 cells (Figs. 5*C* and S5*B*). SRD-15 cells partly retain the response to 25-HC presumably because the cells express residual levels of Insig-2 (Fig. S5*B*) (27). These results indicate that endogenous 25-HC suppresses SREBP-2 activity in an Insig-dependent manner.

We recently reported that the activation of activating transcription factor 4 (ATF4) by exogenous oxysterols requires Insig proteins (28). Next, we tested whether CH25H expression activates the ATF4 axis using the CHO-CH25H^tet-on^ cells. The results show that the induction of CH25H expression is accompanied by the increase in the expression of the ATF4 target genes *Chac1* and *Trb3* in a Dox concentration–dependent manner (Fig. S6). Taken together, these results indicate that Insigs are primary mediators for these cellular responses to 25-HC production.

### Endogenous oxysterols stabilize Insig proteins

**Figure 6:**
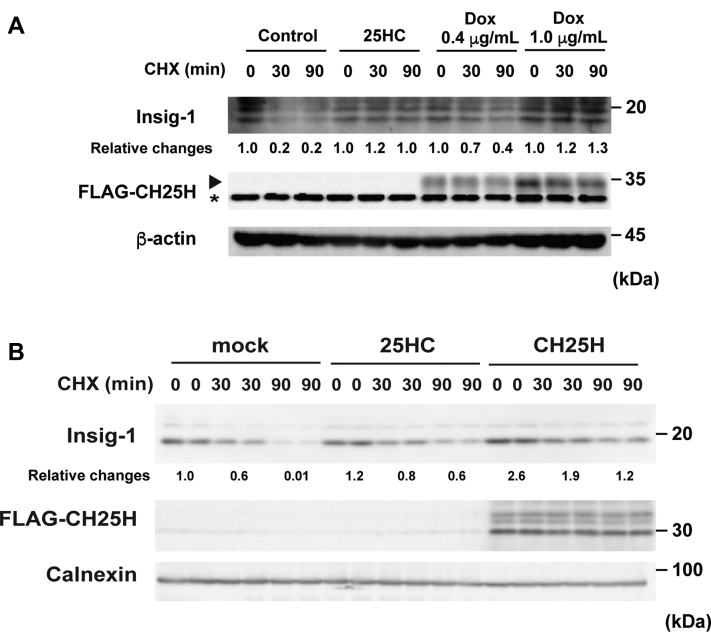
**Endogenous oxysterols stabilize Insig-1 proteins.***A*, expression level–dependent effect of CH25H on Insig-1 stabilization. CHO-CH25H^tet-on^ cells were incubated with or without Dox (0.4 or 1 μg/ml) for 24 h, followed by the treatment with or without CHX (50 μM) and 25-HC (2.5 μM) for 30 or 90 min as indicated. Insig-1 stabilization was assessed by immunoblotting with β-actin as an internal control. Relative changes from two experiments in Insig-1 protein are indicated at the *bottom*. The *asterisk* denotes a nonspecific band. *B*, stabilization of Insig-1 by CH25H expression in HEK293T cells. HEK293T cells were transfected with pFLAG-CH25H (1.0 μg/well). Cells were then treated without or with CHX (50 μM) and 25-HC (2.5 μM) for 30 or 90 min as indicated. Stabilization of Insig-1 was examined by immunoblotting. Calnexin was used as a loading control. Relative changes in Insig-1 protein are indicated at the *bottom*. CHO, Chinsese hamster ovary; CHX, cycloheximide; Dox, doxycycline; 25-HC, 25-hydroxycholesterol; HEK293T, human embryonic kidney 293T cell line; Insig, insulin-induced gene.

Oxysterols can bind Insig proteins and protect them from proteasomal degradation when added exogenously, thereby inhibiting SREBP-2 processing (13, 29). Therefore, we sought to directly determine whether endogenous side-chain oxysterols can also stabilize Insig proteins. The stabilization of Insig was assessed in CHO-CH25H^tet-on^ and HEK293 cells using the translation inhibitor cycloheximide. As reported (29), Insig-1 protein levels were rapidly decreased within 90 min by the cycloheximide treatment in CHO-CH25H^tet-on^ cells not treated with Dox and HEK293 cells by 80% and greater than 90%, respectively, and the reduction was partly retarded by adding 25-HC exogenously (Fig. 6, *A* and *B*). Similar to 25-HC addition, CH25H expression also attenuated Insig-1 degradation, indicating that Insig-1 is also stabilized by endogenous 25-HC as efficiently as exogenous 25-HC. In CHO-CH25H^tet-on^ cells, the rate of Insig-1 stabilization correlated to CH25H expression levels (Fig. 6*A*), suggesting the 25-HC production–dependent inhibition of degradation. Furthermore, in addition to exogenous 27-HC, the expression of CYP27A1 also inhibited the degradation of Insig-1 in CHO cells (Fig. S7). Together, these results indicate that endogenous side-chain oxysterols inactivate SREBP-2 by suppressing proteasomal degradation of Insig-1.

### Quantitative determination of oxysterols produced by cholesterol hydroxylases

**Figure 7:**
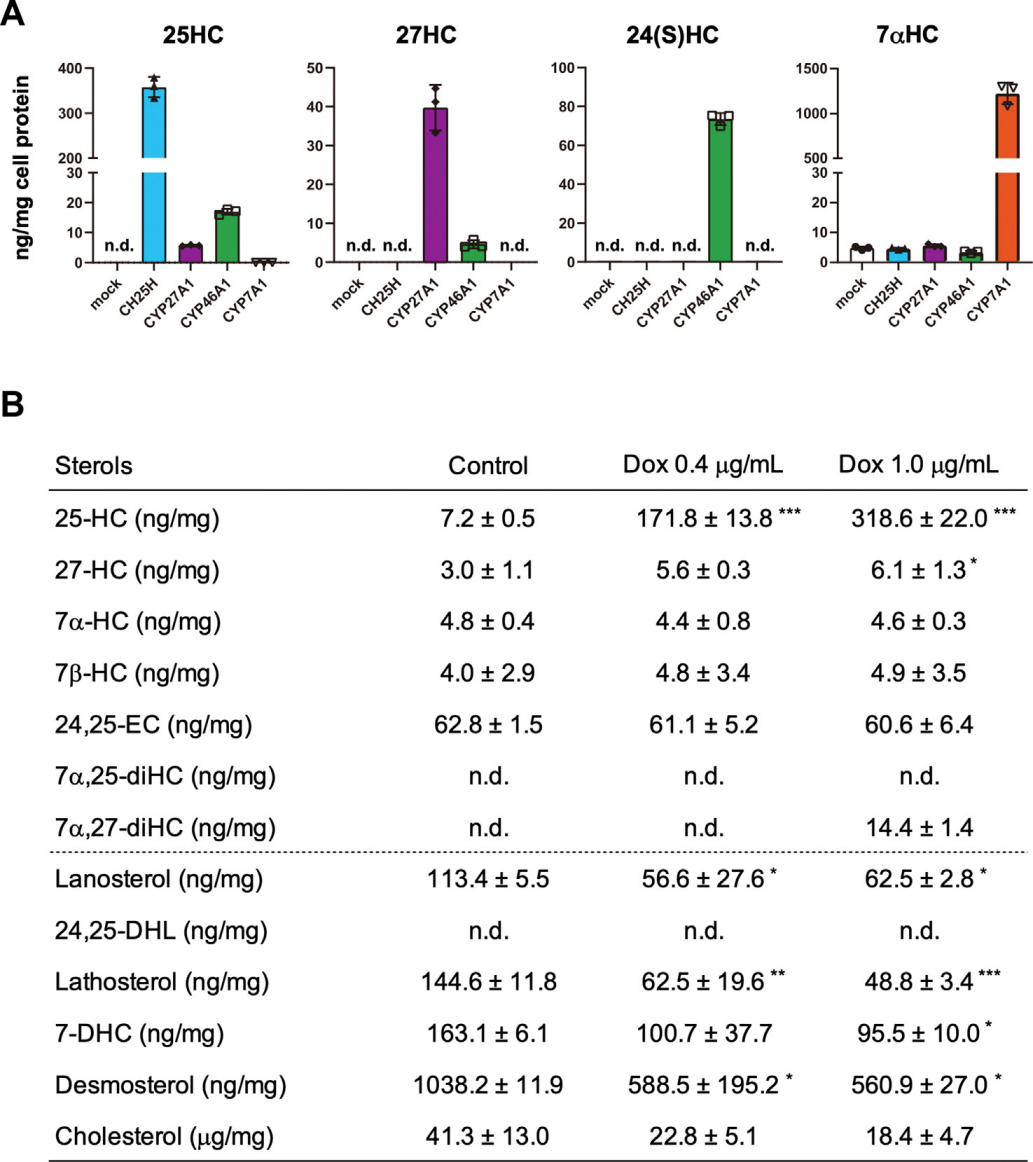
**Quantitative determination of oxysterols synthesized by the expression of cholesterol hydroxylases.***A*, production of oxysterols by the expression of CH25H, CYP27A1, CYP46A1, and CYP7A1. FLAG-CH25H, CYP27A1-FLAG, CYP46A1-FLAG, or CYP7A1-FLAG was expressed in CHO-K1 cells. Twenty-four hours after transfection, cellular lipids were extracted and subjected to GC–MS/MS analysis as described in the Experimental procedures section. The amounts of each sterol were normalized by cell protein. See also Table S1. Data represent means ± SD from three biological replicates. *B*, effect of CH25H expression levels on cellular sterol composition. CHO-CH25H^tet-on^ cells were untreated or treated with different concentrations of Dox (0.4 or 1 μg/ml) for 24 h. Sterol contents were determined by GC–MS/MS. Data represent means ± SD from three biological replicates. Statistical analyses were performed by one-way ANOVA with Dunnett post hoc test (∗*p* < 0.05, ∗∗*p* < 0.01, and ∗∗∗*p* < 0.001). CHO, Chinese hamster ovary cell; 24,25-DHL, 24,25-dihydrolanosterol; 7-DHC, 7-dehydrocholesterol; Dox, doxycycline; 24,25-EC, 24,25-epoxycholesterol; nd, not detected.

Cholesterol hydroxylases catalyze the addition of a hydroxyl group to cholesterol. However, their specificity is not fully validated quantitatively in living cells. Therefore, we finally sought to determine the ability and specificity of CH25H, CYP27A1, CYP46A1, and CYP7A1 to produce oxysterols. Cellular lipids were extracted from CHO-K1 cells expressing either one of four hydroxylases, and nonsaponifiable lipids (containing sterols) were subjected to GC–MS/MS analysis (Fig. 7*A* and Table S1). The results show that the expression of CH25H leads to the robust production of 25-HC but not of other oxysterols, indicating the high specificity of this enzyme to catalyze cholesterol hydroxylation at the C25 position. CYP27A1 expression predominantly generated 27-HC and small amounts of 25-HC. Only 12.5% of oxysterol synthesized by CYP27A1 was 25-HC. 24S-HC was the major oxysterol produced by CYP46A1 expression. Its expression also produced small amounts of 25-HC and 27-HC, accounting for 17.9% and 4.8% of oxysterols produced, respectively. CYP7A1 expression specifically produced 7α-HC. On the other hand, the addition of each oxysterol (2.5 μM) into the culture medium robustly increased cellular amounts of corresponding oxysterols much more than endogenous synthesis by forced expression of each hydroxylase (Table S1). 7α,25-diHC and 7α,27-diHC were as low as undetectable levels in CHO-K1 cells, indicating poor CYP7B1 activity.

Next, we examined the CH25H level–dependent production of 25-HC using the CHO-CH25H^tet-on^ cells. The results show that the cellular amounts of 25-HC correlate to the amounts of Dox added (Fig. 7*B*). The addition of 0.4 and 1.0 μg/ml of Dox increased cellular 25-HC contents by 24 and 44 times, respectively, compared with cells not treated with Dox. Consistent with Figure 7*A*, cellular contents of other oxysterols were not altered by CH25H expression, except for a slight increase in 27-HC. The amounts of intermediate sterols were also measured to ensure suppression of SREBP-2 by CH25H expression. The results showed that CH25H expression causes the reduction of intermediate sterols, including lanosterol, lathosterol, 7-dehydrocholesterol, and desmosterol. Thus, it was demonstrated that CH25H expression leads to the production of 25-HC, which then inhibits SREBP-2 activity and cholesterol biosynthesis.

## Discussion

Supraphysiological levels of oxysterols added exogenously can regulate both SREBP-2 and LXR activities in cultured cells (13, 14). However, oxysterols are present in the circulation and tissues at low levels (15, 19). Studies show that oxysterol synthesis is tightly regulated at transcriptional levels (30, 31). Whether oxysterols, particularly side-chain oxysterols, endogenously synthesized within cells provoke the same cholesterol homeostatic responses as exogenous oxysterols remains largely unknown. In this work, we first carefully determined the sensitivity of SREBP-2 and LXR to a series of oxysterols at various concentrations and found that SREBP-2 was much more sensitive to 25-HC and 27-HC than LXR in CHO cells; in the case of 25-HC, 20 to 100 nM was sufficient to inactivate SREBP-2, whereas 500 nM or higher concentrations were required for LXR activation. Side-chain oxysterols could activate LXR at higher concentrations without adding RXR ligands, confirming that these oxysterols serve as LXR ligands (14). Similar results were obtained with primary hepatocytes; 25-HC markedly suppressed SREBP-2 response gene expression in PRHs. Since LXR activation in hepatocytes requires SREBP-2-dependent sterol synthesis (20, 21), 25-HC also repressed the expression of *Srebf1c*, a primary LXR target in the liver, in these cells. These results indicate that side-chain oxysterols preferentially suppress cholesterol synthesis by inactivating SREBP-2 rather than LXR activation. We next examined whether endogenous oxysterols regulate SREBP-2 and LXR by expressing CH25H, CYP27A1, CYP46A1, or CYP7A1. The results revealed that endogenous side-chain oxysterols, at least those synthesized by these hydroxylases, selectively regulate the SREBP-2 pathway and do not serve as preferred ligands for LXR. Similar results were obtained in macrophages by inducing CH25H expression physiologically; LPS-dependent upregulation of CH25H expression and 25-HC synthesis primarily regulate the SREBP-2 pathway. This is the first to comprehensively examine the effects of endogenous and exogenous oxysterols on cholesterol homeostatic responses in a single-cell system and to confirm these results in more physiological settings. Our results have also uncovered the hydroxylation site–specific and production-dependent roles of oxysterols in cellular cholesterol homeostasis.

**Figure 8:**
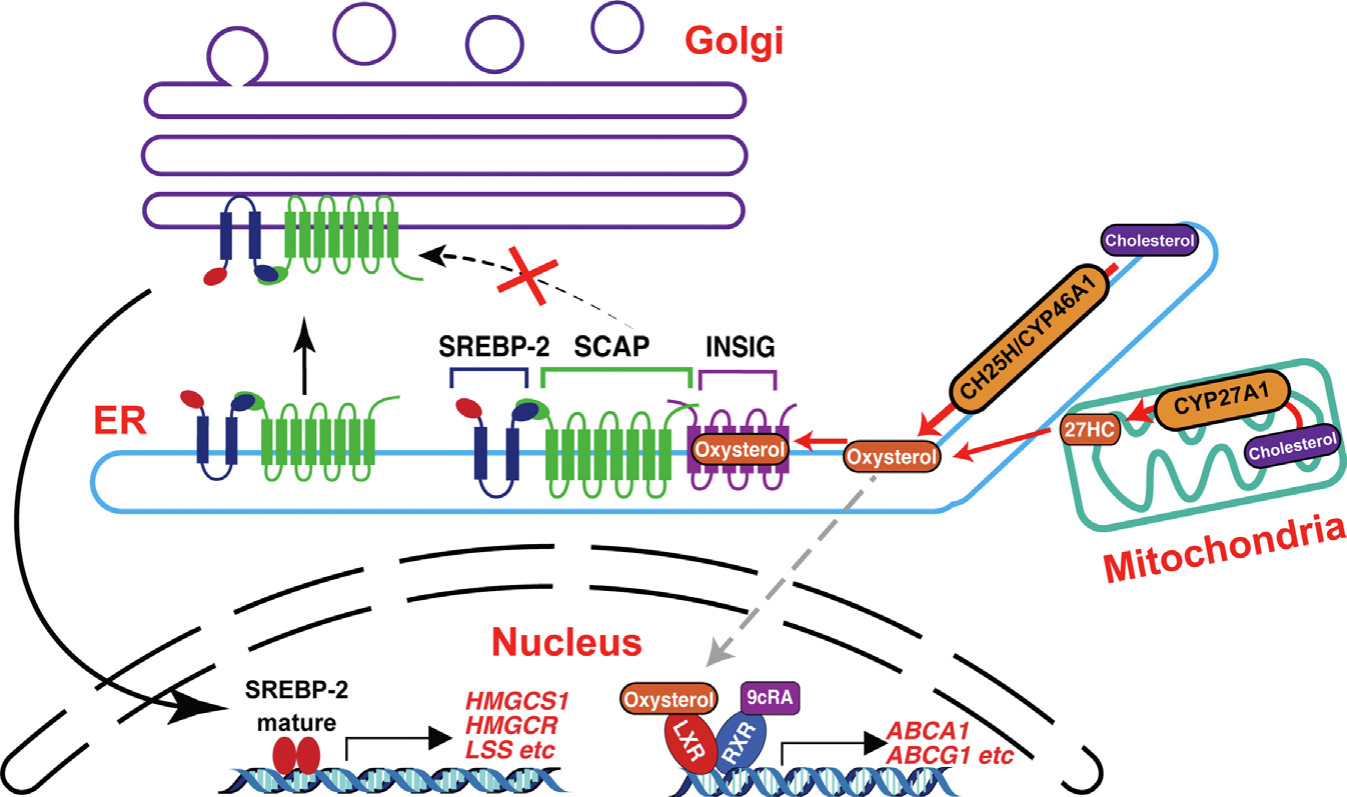
**Model of cholesterol homeostatic regulation by endogenous oxysterols.** Side-chain oxysterols (25-HC, 24S-HC, and 27-HC) synthesized in the ER and mitochondria rapidly bind Insig proteins and inactivate SREBP-2 pathway. Endogenous side-chain oxysterols are poorly available for LXR as its ligand. See text for more details. ER, endoplasmic reticulum; 25-HC, 25-hydroxycholesterol; 27-HC, 27-hydroxycholesterol; 24S-HC, 24S-hydroxycholesterol; Insig, insulin-induced gene; LXR, liver X receptor; SREBP-2, sterol regulatory element–binding protein-2.

How do *de novo* synthesized oxysterols regulate cholesterol homeostatic responses? CH25H and CYP46A1 localize to the ER and synthesize 25-HC and 24S-HC, respectively, in the ER membrane (17), where SREBP, SCAP, and Insig form a trimeric complex (1). CYP27A1 resides in the inner mitochondrial membrane and catalyzes the conversion of cholesterol into 27-HC with the function of StAR (17). The mitochondria often form membrane contact sites with the ER, which facilitates the exchange of lipids between these two organelles (32). Thus, 27-HC produced in the mitochondria may efficiently be transported to the ER. Accordingly, these three side-chain oxysterols could be enriched in the ER membrane and rapidly bind Insig to prevent its degradation, which in turn suppresses the translocation of the SREBP2–SCAP complex to the Golgi (Fig. 8). On the other hand, the current results have shown that the expression of enzymes catalyzing cholesterol side-chain hydroxylation and subsequent biosynthesis of corresponding oxysterols do not lead to the activation of LXR, indicating that side-chain oxysterols synthesized in the ER and mitochondria are poorly available for LXR. The majority of LXR localizes in the nucleus. Thus, we hypothesize that endogenous oxysterols hardly enter the nuclei, or other more potent endogenous LXR ligand exists as suggested previously (21). In contrast, exogenous side-chain oxysterols are capable of inducing LXR target gene expression at higher concentrations. Previous studies showed that in addition to the nucleus, LXR localizes to the plasma membrane (PM) where it interacts with ABCA1 (33), a prominent LXR target. Oxysterols added exogenously enter cells from the PM. Therefore, they could promptly interact with and activate PM-localized LXR than those synthesized intracellularly. Moreover, cholesterol homeostatic responses to side-chain oxysterols could be differentially regulated by the abundance and/or activity of Insig, LXR, and CYP7B1 under various physiological and pathophysiological settings. Further studies are required to clarify how intracellular distribution and transport of oxysterols are regulated, how the expression of different oxysterol effector and modulator proteins is controlled, and whether a more preferential endogenous LXR ligand exists.

In analogy to our findings, whether side-chain oxysterols serve as endogenous LXR ligands remains controversial. In *Ch25h*/*Cyp27a1*/*Cyp46a1* triple KO mice, the expression of some LXR target genes in the liver was poorly induced when mice were fed a cholesterol-rich diet (34). However, the effect of the deletion of these three hydroxylase genes on LXR was relatively modest. Furthermore, cholesterol loading of macrophages from triple KO mice can markedly upregulate the expression of LXR target genes (35). Among side-chain oxysterols, 24S-HC is one of the most potent LXR ligands in cultured cells (14, 36). However, overexpression of CYP46A1 in mice showed little effect on LXR target gene expression despite the accumulation of 24S-HC in tissues and blood (37). Similarly, in *CYP27A1* transgenic mice and *Cyp7b1*^−/−^ mice where 27-HC, and 25-HC and 27-HC are markedly elevated, respectively, in blood and liver, the upregulation of LXR target genes in the liver was undetectable (38). All these results indicate that the increase in side-chain oxysterols is insufficient to activate LXR *in vivo*. Similar observations were reported for SREBP-2 regulation *in vivo*. Both in *CYP46A1* and *CYP27A1* transgenic mice, SREBP-2 target gene expression is not downregulated in the liver (37, 38). Furthermore, in *Cyp7b1*^−/−^ mouse livers, the expression of SREBP-2 target genes and the rate of cholesterol biosynthesis are largely unaffected (38, 39). These results may support the view that 25-HC, 27-HC, and 24S-HC play only an auxiliary role in cholesterol homeostatic responses in the liver. However, SREBP-2-dependent sterol synthesis is required for the expression of the LXR target gene *Srebp-1c*, but not *Abca1*, in mouse livers (21), suggesting that a certain sterol(s) serves as an endogenous LXR ligand. Our results with PRHs also support this view. On the other hand, recent studies have identified CH25H as an infection-inducible gene (30). LPS and interferon markedly increase CH25H expression and 25-HC production in immune cells, including macrophages (24, 40, 41). CH25H-dependent synthesis of 25-HC protects cells from virus infection by inhibiting virus replication and fusion and exerts anti-inflammatory responses (30). These effects depend on the suppression of SREBP-2 activity and cholesterol biosynthesis (42, 43). Our results showed that LPS-dependent 25-HC production primarily regulates SREBP-2 in murine macrophages. All these observations indicate that 25-HC-dependent inactivation of SREBP-2, not LXR activation, plays a crucial role in immune response both *in vitro* and *in vivo*.

In addition to side-chain oxygenated cholesterol (such as 25-HC, 27-HC, and 24S-HC), desmosterol and 24,25-epoxycholesterol (24,25-EC) can serve as LXR ligands (44, 45). Desmosterol is an intermediate sterol, whereas 24,25-EC is synthesized from dioxidosqualene by the shunt pathway or directly from desmosterol by CYP46A1 (7, 16). Our findings showed that in CHO cells, CH25H expression reduced cellular desmosterol contents, which is consistent with the suppression of SREBP-2. Furthermore, our data showed that CYP46A1 expression did not increase cellular amounts of 24,25-EC, suggesting that CYP46A1 plays a minor role in the conversion of desmosterol to 24,25-EC in living cells, at least in CHO cells although this enzyme has the 24,25-epoxidation activity on desmosterol *in vitro* (46). Therefore, it is conceivable that the expression of cholesterol side-chain hydroxylases suppresses SREBP-2 activity and reduces cellular contents of desmosterol and/or 24,25-EC. In line with this view, the HMGCR inhibitor statins suppress LXR activity (47). Our results thus suggest that desmosterol and 24,25-EC may not serve as preferred ligands for LXR in a context where cholesterol side-chain hydroxylase expression is upregulated. Therefore, we speculate that desmosterol serves as an endogenous LXR ligand when cholesterol biosynthesis is activated. This hypothesis may be supported by our previous findings that ABCA1 releases not only cholesterol but also intermediate sterols such as lanosterol (48). Since lanosterol is more cytotoxic than cholesterol and is a poor substrate for ACAT1, desmosterol-dependent upregulation of ABCA1 may provide cells with means to eliminate excess cytotoxic intermediate sterols. In addition, it has been shown that desmosterol accumulates in macrophage foam cells within atherosclerotic lesions (49, 50) and activates LXR (35).

In conclusion, we disclosed the role of endogenously synthesized side-chain oxysterols in cellular cholesterol homeostasis; they selectively suppress SREBP-2 activity by protecting Insig proteins from their proteasomal degradation without serving as LXR ligands. Moreover, this study is the first to comprehensively and quantitatively identify oxysterols produced by the four major cholesterol hydroxylases, CH25H, CYP27A1, CYP46A1, and CYP7A1, in living cells.

## Experimental procedures

### Materials

Sterols were obtained from commercial sources as follows: cholesterol (C8667), 24(S)-hydroxycholesterol (SML1648), 25-hydroxycholesterol (H1015), 27-hydroxycholesterol (SML2042), 7α,25-diHC (SML0541), 7-dehydrocholesterol (30800), and 7β-hydroxycholesterol (H6891) from Sigma; desmosterol (NS460402), lanosterol (NS460102), lathosterol (NS460502), and 24,25-dihydrolanosterol (NS460201) from Nagara Science; 7α,27-diHC (700024P), 7α-hydroxycholesterol (700034P), cholesterol-d7 (700041P), 25-hydroxycholesterol-d6 (LM4113-1EA) from Avanti Polar Lipids; and 24,25-EC (Ab141633) from Abcam. T0901317 and Kdo2-Lipid A (699500P) were from Sigma and Avanti Polar Lipids, respectively.

### Cell culture

CHO-7 and SRD-15 cells (27) were isolated in the laboratories of Drs Joseph Goldstein and Michael Brown (UT Southwestern Medical Center) and Dr Russell DeBose-Boyd (UT Southwestern Medical Center), respectively. CHO-K1 and 25RA cells (51) were kind gifts of Dr Ta-Yuan Chang (Geisel School of Medicine at Dartmouth). All the CHO cell lines were maintained in Dulbecco's modified Eagle's medium (DMEM)/F12 1:1 mixture supplemented with 7.5% fetal bovine serum (FBS) and penicillin/streptomycin. A2058 (obtained from JCRB Cell Bank) and HEK293T cells were cultured in DMEM with 10% FBS and penicillin/streptomycin. J774.1 cells (RIKEN Cell Bank) were maintained in RPMI1640 medium supplemented with 10% FBS and penicillin/streptomycin. PRHs were isolated from nonfasted male Sprague–Dawley rats at 8 to 10 weeks old (Japan Clea) as described previously (52) with minor modifications. Rats were housed in a 12 h light/12 h dark schedule at 23 ± 2 °C and fed ad labium with a standard chow diet (Labo MR Stock; Nosan Corporation) and water. Animal experiments were approved by the Animal Care and Use Committee of the University of Tokyo and were conducted according to the guidelines of the Animal Care and Use Committee of the University of Tokyo. After rats were anesthetized with isoflurane, liver was perfused with perfusion buffer (Hanks' balanced salt solution with 1 mM EGTA) *via* portal vein and then perfused with digestion buffer (Hanks' balanced salt solution with 10 mM Hepes, 4 mM NaHCO_3_, and 0.2% collagenase [Sigma]). Afterward, liver was taken, the hepatic capsule was stripped with tweezers in digestion buffer, and dissociated cells were collected into a tube by passing through a 100 μm cell strainer (Corning). Cells were pelleted by centrifugation at 500 rpm for 1 min at 4 °C. They were then mixed with Percoll (Cytiva), and dead cells were removed by centrifugation at 600 rpm for 10 min at 4 °C. Cells were seeded into collagen I-coated 12-well plates (Corning; catalog no.: 356500) at a density of 5 × 10^5^ cells/well and incubated for 4 to 5 h in William’s medium E (Sigma) supplemented with 7.5% FBS, 10 nM dexamethasone, 2 mM l-glutamine, and penicillin/streptomycin. The attached cells were gently washed once with warmed PBS and further incubated in DMEM containing 7.5% FBS and penicillin/streptomycin overnight before treatment. All cells were cultured in a humidified incubator at 37 °C with 5% CO_2_.

### Plasmid constructs

The coding sequence of human *CH25H* was amplified from human genomic DNA (because human *CH25H* does not contain any introns) and cloned into p3×FLAG-CMV-10 expression vector (Sigma) at the BamHI/EcoRI site to generate pFLAG-CH25H. pFLAG-CH25H was used as a template to create the active site mutant CH25H (CH25H^H242Q/H243Q^) expression plasmid. The 3×FLAG-CH25H sequence was amplified using pFLAG-CH25H as a template and cloned into pTetOne vector (Clontech) at the BamHI/MluI site to generate pFLAG-CH25H^tet-on^. The coding sequence of human *CYP27A1*, *CYP7A1*, and *CYP46A1* was amplified by PCR and cloned into p3×FLAG-CMV-14 expression vector (Sigma) at EcoRV/XbaI, HindIII/BamHI, and EcoRI/BamHI sites, respectively. These CYP27A1-FLAG, CYP7A1-FLAG, and CYP46A1-FLAG expression constructs were referred to as pCYP27A1-FLAG, pCYP7A1-FLAG, and pCYP46A1-FLAG, respectively. Complementary DNA (cDNA) for human StAR was cloned into p3×FLAG-CMV-14 expression vector as described previously. Primers used for cloning are listed in Table S2.

### Transfection and isolation of stable transfectants

Cells were seeded into 6-well plates. About 18 to 24 h later, cells were transfected with plasmids using Lipofectamine LTX Reagent (Thermo Fisher Scientific) according to the manufacturer’s protocol. Polyethylenimine Max (Polysciences) was used for plasmid transfection in HEK293T cells. Twenty-four to 48 h after transfection, cells were used for further experiments.

For the isolation of CHO-K1 cells harboring pFLAG-CH25H^tet-on^ (CHO-CH25H^tet-on^ cells), CHO-K1 cells seeded into 6-well plate were cotransfected with pFLAG-CH25H^tet-on^ (2 μg/well) and pMAM-BSD (0.1 μg/well). Twenty-four hours after transfection, cells were grown in medium containing blasticidin (7 μg/ml) for 5 days. Blasticidin-resistant clones were isolated by limited dilution, and clones that were positive for Dox-dependent FLAG-CH25H expression were selected by immunoblotting with anti-FLAG antibody.

### RNA isolation and quantitative PCR

Total RNA was isolated using ISOGEN II (Nippon Gene). cDNA was then reverse synthesized using total RNA (2 μg/reaction) isolated and High-Capacity cDNA Reverse Transcription Kit (Applied Biosystems). mRNA levels of a gene were analyzed by quantitative real-time PCR using FastStart Universal SYBER Green Maser (ROX) (Roche) and a specific primer set (Table S3). Quantitative PCR was performed using a Quant Studio 6 Flex Real-Time PCR System or a StepOnePlus instrument (Applied Biosystems). mRNA levels were normalized to *18S* ribosomal RNA levels.

### Luciferase reporter assay

CHO-K1 cells were plated into 12-well plate and grown for overnight. On day 1, pGL2 containing hamster *Hmgcs1* promoter (0.25 μg/well), pCMV-β-galactosidase (0.25 μg/well) (for normalization), and plasmids of interest (0.5 μg/well) were transfected using Lipofectamine LTX. Five hours after transfection, medium was switched to medium supplemented with 0.1% FBS, and cells were further incubated for 16 h. Afterward, cells were lysed with lysis buffer (25 mM Tris phosphate [pH 7.8], 2 mM DTT, 2 mM EDTA, 10% [v/v] glycerol, and 1% [v/v] Triton X-100), and cell lysate was subjected to luciferase assay. The luciferase activity was measured using a Lumat LB9508 luminometer (Berthold). β-galactosidase activity was also measured using a SpectraMax M2e (Molecular Devices) at 415 nm for normalization.

### Immunoblotting

Cell lysate was prepared using urea buffer (50 mM Tris–HCl [pH 8.0], 50 mM sodium phosphate [pH 8.0], 100 mM NaCl, 8 M urea, 0.5% Protease inhibitor cocktail [Nacalai]) or radioimmunoprecipitation assay buffer (50 mM Tris–HCl [pH 7.4], 150 mM NaCl, 1 mM EDTA, 1% NP-40, 0.25% sodium deoxycholate, and 0.5% Protease inhibitor cocktail) as described (53). After determination of protein concentration using bicinchoninic acid assay (Thermo Fisher), equal amounts of proteins were subjected to SDS-PAGE and immunoblot analysis. Primary antibodies used are as follows; anti-Insig1 polyclonal antibody (PAB8786) from Abnova, anti-FLAG monoclonal antibody (clone M2; F3165) and anti-β-actin monoclonal antibody (clone AC15; A1978) from Sigma, anti-ABCA1 polyclonal antisera (54) (gift from Dr Shinji Yokoyama, Chubu University), anti-SREBP-2 monoclonal antibody (clone 7D4) hybridoma supernatant and anti-HMGCR monoclonal antibody (clone A-9) hybridoma supernatant (gift from Dr Ta-Yuan Chang, Geisel School of Medicine at Dartmouth). Band intensities were quantified using ImageJ software (National Institute of Health) or Evolution Capt software (Vilber). Protein expression was normalized to β-actin expression unless specified in figure legends.

### Immunostaining and immunofluorescence microscopy

Cells were seeded onto a 18 mm × 18 mm glass coverslip (Matsunami) placed in a 6-well plate and grown overnight. Cells were then transfected with plasmid as specified in figure legends. Afterward, cells were fixed with 4% paraformaldehyde in PBS for 10 min and permeabilized with 0.1% Triton X-100 for 5 min at room temperature (55). After blocking with 5% FBS in PBS for 1 h, cells were incubated with primary antibodies (anti-Tom20 monoclonal antibody from Cell Signaling Technology, catalog no.: 42406, anticalnexin monoclonal antibody from Cell Signaling Technology, catalog no.: 2679, or anti-FLAG monoclonal antibody from Sigma, F3165) for 1 h. After washing with 1% FBS in PBS three times, specimens were incubated with Alexa Fluor 488–conjugated antimouse immunoglobulin G (Invitrogen; A11029) and Alexa Fluor 568–conjugated anti-rabbit immunoglobulin G (Invitrogen; A11036) (1:800 dilution) in 2% FBS in PBS for 45 min. Nuclei were stained with 4′,6-diamidino-2-phenylindole. Specimens were washed with PBS for three times and mounted with ProLong Diamond Antifade Mountant (Thermo Fisher Scientific). Immunofluorescence confocal images were acquired using a Zeiss LSM800 with Airyscan equipped with a Plan-Apochromat 63×/1.40 Oil DIC M27 objective (Carl Zeiss). Images were processed with a Zen software (Carl Zeiss) and Fiji software (Fiji).

### GC–MS/MS analysis

Cellular lipids were extracted with hexane/2-propanol (3:2, v/v) containing 0.01% dibutyl hydroxytoluene and collected into a glass tube. Cholesterol-d7 (100 ng/tube) and 25-hydroxycholesterol-d6 (10 ng/tube) were added to the sample as internal controls. After evaporation under nitrogen gas stream, 1 ml of 100% EtOH and 300 μl of 10 N KOH (in 75% EtOH, v/v) was added to each tube, and lipids were saponified at 80 °C for 1 h. Afterward, nonsaponified lipids were extracted with chloroform and dried under nitrogen gas. The sample was then derivatized in 100 μl of 1:1 pyridine/*N*-methyl-*N*-trimethylsilyl trifluoroacetamide (GL Science) at 80 °C for 1 h and transferred to a new glass vial for GC–MS/MS analysis. Cellular protein was solubilized in 0.1 N NaOH to measure protein contents using bicinchoninic acid assay.

GC–MS/MS analysis was performed using GCMS-TQ8040 NX (Shimadzu) equipped with an AOC-20i autosampler (Shimadzu) and a BPX5 GC column (30 m × 0.25 mm, 0.25 μm; TRAJAN). One microliter of sample was injected using the autosampler in splitless mode. The temperature of the sample injection port was set at 275 °C, and helium was flowed at 49.5 ml/min as carrier gas. The oven temperature was set at 200 °C and kept for 1 min. Afterward, the temperature was increased by 25 °C/min to 250 °C, 15 °C/min to 290 °C, and 5 °C/min to 320 °C, and 320 °C was kept for 2 min. Detection by MS/MS was performed using monitoring reaction mode. The retention time, quantification ion, confirmation ion, and collision energy for each sterol were summarized in Table S4. Cholesterol-d7 and 25-hydroxycholesterol-d6 were used as internal controls for nonhydroxylated sterols and hydroxylated sterols, respectively. Sterol contents were normalized to cell protein.

### Statistical analysis

Data are presented as mean ± SD from at least three independent biological replicates. All experiments were repeated on two or more occasions with similar results. Statistical analyses were performed using Student’s *t* test or one-way ANOVA with Dunnett or Tukey–Kramer post hoc test as specified in figure legends with RStudio software (Posit). *p* < 0.05 was considered statistically significant.

## Data availability

All data are contained in the article and supporting information.

## Supporting information

This article contains supporting information.

## Conflict of interest

The authors declare that they have no conflicts of interest with the contents of this article.
