# Supplementary material for: Hydroxylation site–specific and production-dependent effects of endogenous oxysterols on cholesterol homeostasis: Implications for SREBP-2 and LXR
Source: J Biol Chem. 2022 Nov 22;299(1):102733. doi: 10.1016/j.jbc.2022.102733 (PMC9792893; doi:10.1016/j.jbc.2022.102733)
Supplement: Supplemental Figures and Tables [file mmc1.pdf]

Supporting information

Hydroxylation site-specific and production-dependent effects of endogenous oxysterols on cholesterol homeostasis: Implications for SREBP-2 and LXR

Hodaka Saito[1], Wakana Tachiura[1], Mizuki Nishimura[1], Makoto Shimizu[2], Ryuichiro Sato[1,2,3], Yoshio Yamauchi[1,2,3]*

[1]Laboratory of Food Biochemistry, Department of Applied Biological Chemistry, Graduate School of Agricultural and Life Sciences, The University of Tokyo, Tokyo, Japan
[2]Nutri-Life Science Laboratory, Department of Applied Biological Chemistry, Graduate School of Agricultural and Life Sciences, The University of Tokyo, Tokyo, Japan
[3]AMED-CREST, Japan Agency for Medical Research and Development, Tokyo, Japan

*Corresponding author:
Yoshio Yamauchi, Ph.D.
Department of Applied Biological Chemistry, Graduate School of Agricultural and Life Sciences, The University of Tokyo
1-1-1 Yayoi, Bunkyo-ku, Tokyo, Japan
Email: yoshio-yamauchi@g.ecc.u-tokyo.ac.jp

Supporting Tables: Table S1 – S4
Supporting Figures: Figure S1 – S7

Table S1

Oxysterol contents in CHO-K1 cells ectopically expressed the indicated hydroxylases or incubated with the indicated oxysterols at 2.5 μM for 16 hr. Part of the results are also presented in Figure 7A.

Sterols	mock	CH25H	CYP27A1	CYP46A1	CYP7A1	25HC	27HC	24(S)HC	7αHC
25HC (ng/mg)	n.d.	357.8 ± 18.3	5.7 ± 0.2	17.0 ± 0.8	0.7 ± 0.9	780.0 ± 281.0	3.3 ± 0.5	1.1 ± 0.2	3.3 ± 0.4
27HC (ng/mg)	n.d.	n.d.	39.8 ± 4.8	4.6 ± 0.8	n.d.	n.d.	350.6 ± 75.8	6.3 ± 0.2	5.0 ± 0.2
24(S)HC (ng/mg)	n.d.	n.d.	n.d.	73.5 ± 2.6	n.d.	n.d.	n.d.	600.7 ± 38.2	2.1 ± 0.2
7αHC (ng/mg)	4.8 ± 0.4	4.6 ± 0.3	5.6 ± 0.4	3.5 ± 0.3	1223.8 ± 99.0	4.4 ± 0.8	3.9 ± 0.2	3.3 ± 0.4	841.0 ± 90.1
24, 25-EC (ng/mg)	41.0 ± 1.4	52.9 ± 8.7	40.2 ± 2.9	37.5 ± 3.0	17.3 ± 0.7	25.6 ± 0.3	32.2 ± 0.9	36.9 ± 2.0	57.3 ± 1.6

Table S2

Primers used for cloning.

pFLAG-CH25H	FW: 5’-CCGAATTCAAGCTGCCACAACTGCTCCG-3’<br>RV: 5’-CCGGGATCCTCACCGCGCTGGGACAGATGCAGT-3’
pCYP27A1-FLAG	FW: 5’-ATATATGATATCGATGGCTGCGCTGGGCTGCGC-3’<br>RV: 5’-TATATATCTAGAGCACTGTCTCTGCAGGAACTGCA-3’
pCYP7A1-FLAG	FW: 5’-ATATATAAGCTTATGATGACCACATCTTTGATTTGGG-3’<br>RV: 5’-ATATATGGATCCCAAATGCTTGAATTTATATTTAAATTCAAT-3’
pCYP46A1-FLAG	FW: 5’-CGGAATTCATGAGCCCCGGGCTGCTGCTGCT-3’<br>RV: 5’-CGGGATCCGCAGGGGGGTGGTGGGGGTGCGGGCT-3’
pStAR-FLAG	FW: 5’-ATATATAAGCTTATGCTGCTAGCGACATTCAAGCTGTGCGCT-3’<br>RV: 5’-ATATATGAATTCCGACACCTGGCTTCAGAGGCAGGG-3’
FLAG-CH25H$^{tet\text{-}on}$	FW: 5’-ATATATATACGCGTATGGACTACAAAGAC-3’<br>RV: 5’-ATATATGGATCCTCACTACCGCGCTG-3’
FLAG-CH25H$^{H242Q/H243Q}$	FW: 5’-CAACAGGACCTGCATCACTCTCA-3’<br>RV: 5’-CACCACACCCCCGTACCACCC-3’

Table S3

Primers used for qPCR analysis

hamster *18s rRNA*	FW: 5’-TAAGTCCCTGCCCTTTGTACACA-3’<br>RV: 5’-GATCCGAGGGCCTCACTAAAC-3’
hamster *Abca1*	FW: 5’-GCTCTGGTGTTCAGCCTAAT -3’<br>RV: 5’-CTGGTTAGAGCATTCAGGAGTT-3’
hamster *Abcg1*	FW: 5’-GGGATCAGAACAGTCGCCTG-3’<br>RV: 5’-CGAGGTCTCTCTTATAGTCAGCGTC-3’
hamster *Hmgcs1*	FW: 5’-CCTATGACTGCATTGGGCG-3’<br>RV: 5’-CCCAGACTCCTCAAACAGCTG-3’
hamster *Hmgcr*	FW: 5’-CCCAAAGAAAGCTCCAGACA-3’<br>RV: 5’-CACTCTCAGTTTCCACCACTAAT-3’
hamster *Sqs*	FW: 5’-CCCAAGTCCAGTTCTCATCTAC-3’<br>RV: 5’-CCTTCAGGTGGTCAGGTATTT-3’
hamster *Lss*	FW: 5’-GGAGCTCTATGTGGAGGACTAT-3’<br>RV: 5’-CTCAGGCTGGTACTATGGAAAC-3’
hamster *Insig1*	FW: 5’-ATCAACCACGCCAGTGCCAAAT-3’<br>RV: 5’-CGAATGTCCACCACAAGCCCAAAG-3’
hamster *Insig2*	FW: 5’-GGGTGGTGCTCTTCTTCATTG-3’<br>RV: 5’-CAGGTGGAAAAAGTGTCACGTTT-3’
hamster *Fasn*	FW: 5’-CATTCATCAGGCCACCATACT-3’<br>RV: 5’-GTCTTCCCACTGGTACACTTTC-3’
human *CH25H*	FW: 5’-ATCACCACATACGTGGGCTTT-3’<br>RV: 5’-GTCAGGGTGGATCTTGTAGCG-3’
human *HMGCS1*	FW: 5’-GACTTGTGCATTCAAACATAGCAA-3’<br>RV: 5’-CTGTAGCAGGGAGTCTTGGTACT -3’
human *SQS*	FW: 5’-ATGACCATCAGTGTGGAAAAGAAG -3’<br>RV: 5’-CCGCCAGTCTGGTTGGTAA -3’
human *18S rRNA*	FW: 5’-ACCGCAGCTAGGAATAATGGA -3’<br>RV: 5’-GCCTCAGTTCCGAAAACCA -3’
mouse *Ch25h*	FW: 5'-TGCTACAACGGTTCGGAGC-3'<br>RV: 5'-AGAAGCCCACGTAAGTGATGAT-3'
mouse *Il-6*	FW: 5'-TAGTCCTTCTACCCCAATTTCC-3'<br>RV: 5'-TTGGTCCTTAGCCACTCCTTC-3'
mouse *Hmgcr*	FW: 5'-CCGGCAACAAGATCTGTG-3'<br>RV: 5'-ATGTACAGGATGGCGATGCA-3'
mouse *Hmgcs1*	FW: 5'-GCGTCTTTGCTTGTGTCTAATC-3'<br>RV: 5'-GAGAACACTCCAACCCTCTTC-3'

mouse *Sqs*	FW:5'-CACACTGGCTGCCTGTTACAA-3'<br>RV: 5'-CCCCTTCCGAATCTTCACTACTC-3'
mouse *Abca1*	FW: 5’-TCCTCATCCTCGTCATTCAAA-3’<br>RV: 5'-GGACTTGGTAGGACGGAACCT-3'
mouse *Abcg1*	FW: 5'-GCTGTGCGTTTTGTGCTGTT-3'<br>RV: 5'-TGCAGCTCCAATCAGTAGTCCTAA-3'
mouse *18s rRNA*	FW: 5’-ACCGCAGCTAGGAATAATGGA -3’<br>RV: 5’-GCCTCAGTTCCGAAAACCA -3’
rat *Hmgcs1*	FW: 5’-AAAGATGTGGGAATCGTTGC-3’<br>RV: 5’-TGAACCACAGTCAGGCAGAG-3’
rat *Hmgcr*	FW: 5’-TGCCAACTACTTTGTCTTCATGACA-3’<br>RV: 5’-TCACGGCTTTCCCGAGAA-3’
rat *Ldlr*	FW: 5’-GCGTCAGCCGATGCATTC-3’<br>RV: 5’-TAGTTCATCCGAGCCATTTTCAC-3’
rat *Pcsk9*	FW: 5’-CGGGGCTATGTCATCAAGGTT -3’<br>RV: 5’-TGCTCTGGGCGAAGACTAATG -3’
rat *Abca1*	FW: 5’-CCCGGCGGAGTAGAAAGG-3’<br>RV: 5’-AGGGCGATGCAAACAAAGAC-3’
rat *Srebf1c*	FW: 5’-GGAGCCATGGATTGCACATTTG-3’<br>RV: 5’-CAAATAGGCCAGGGAAGTCA-3’

Table S4

Detailed parameters for each sterol in GC-MS/MS analysis

Sterols	Retention time (min)	Quantification		Confirmation			
		MRM transition (m/z)	CE (eV)	MRM transition (m/z)	CE (eV)	MRM transition (m/z)	CE (eV)
7α-Hydroxycholesterol	8.77	456 > 208.3	18	456 > 119	30	456 > 95.1	33
Cholesterol-d7	9.18	336 > 121.1	18	375 > 145.1	18	336 > 109.2	18
Cholesterol	9.23	368 > 145.1	21	329 > 95.1	27	329 > 81.1	24
Desmosterol	9.53	129 > 73	15	129 > 57.9	30	129 > 127.1	15
7-Dehydrocholesterol	9.56	351 > 145.1	30	351 > 128.1	42	-	
Lathosterol	9.71	213 > 157.2	12	213 > 81.1	15	-	
7β-Hydroxycholesterol	9.71	456 > 233.2	18	233 > 73.1	24	-	
7α, 25-Dihydroxycholesterol	10.55	131 > 73.1	18	544 > 73.2	39	-	
Lanosterol	10.70	393 > 95.2	24	393 > 187.4	12	-	
24,25-Epoxycholesterol	10.78	143 > 73.1	18	129 > 73.1	15	143 > 128.1	18
24(S)-Hydroxycholesterol	10.98	159 > 69.1	9	159 > 73.1	18	-	
7α, 27-Dihydroxycholesterol	10.98	103 > 73.1	9	544 > 233.2	33	-	
25-Hydroxycholesterol-d6	11.10	137 > 73.2	15	137 > 58.2	30	-	
25-Hydroxycholesterol	11.16	131 > 73.2	9	131 > 58.1	30	-	
27-Hydroxycholesterol	11.64	129 > 73.1	15	456 > 131.2	42	417 > 69	42

CE: Collision enegy

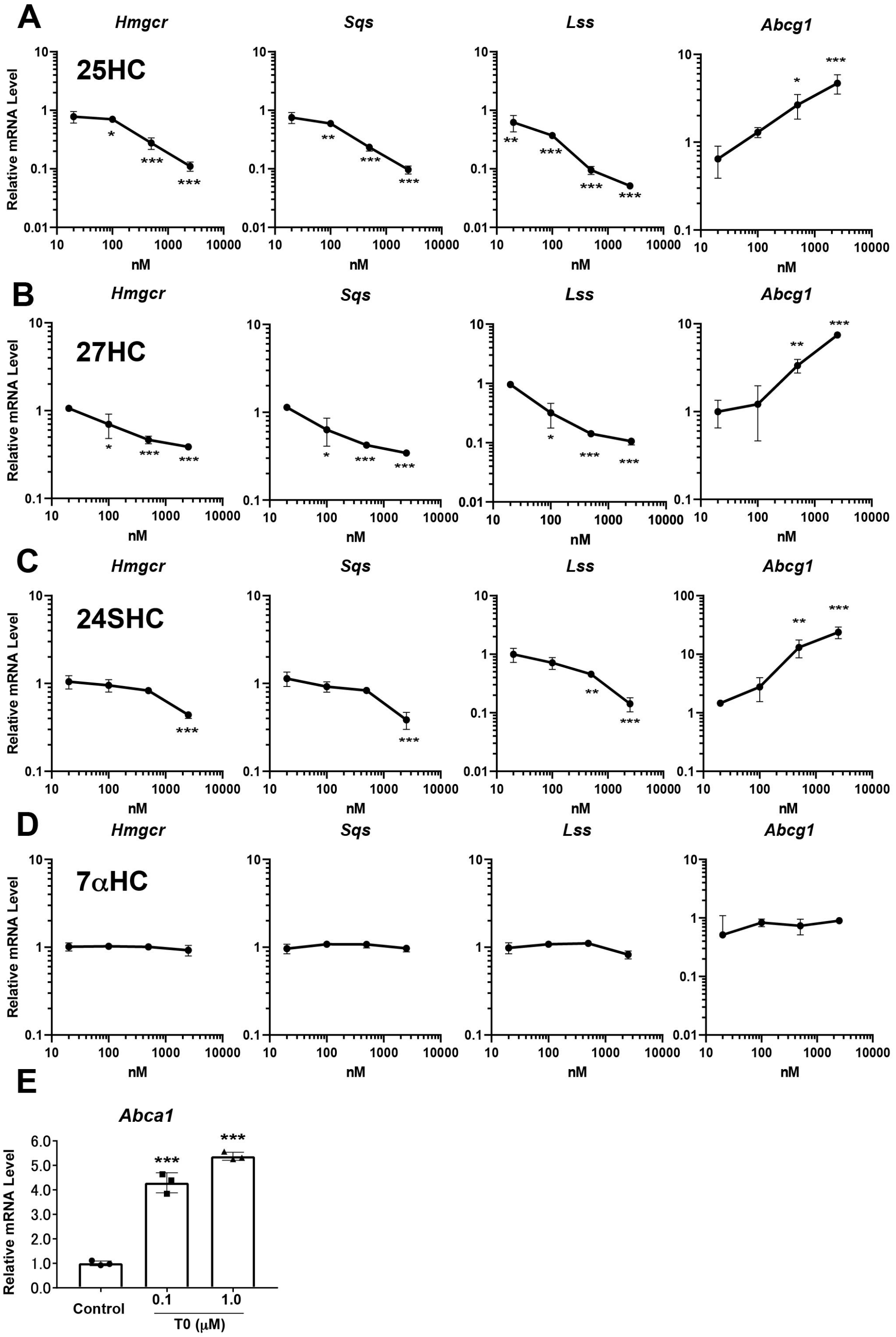

Figure S1. SREBP-2 is more sensitive to exogenous side-chain oxysterols than LXR (Related to Fig. 1).

CHO-K1 were treated without or with 25-HC (A), 27-HC (B), 24S-HC (C), 7α-HC (D) or T0901317 (E), at different concentration for 24 h as in Figure 1. mRNA levels of the indicated genes (A–D) or *Abca1* (E) were measured by qPCR. Data represent means ± SD (n = 3). Statistical analyses were performed by one-way ANOVA with Dunnett post hoc test by comparing to the vehicle treatment group (*p<0.05, **p<0.01, ***p<0.001).

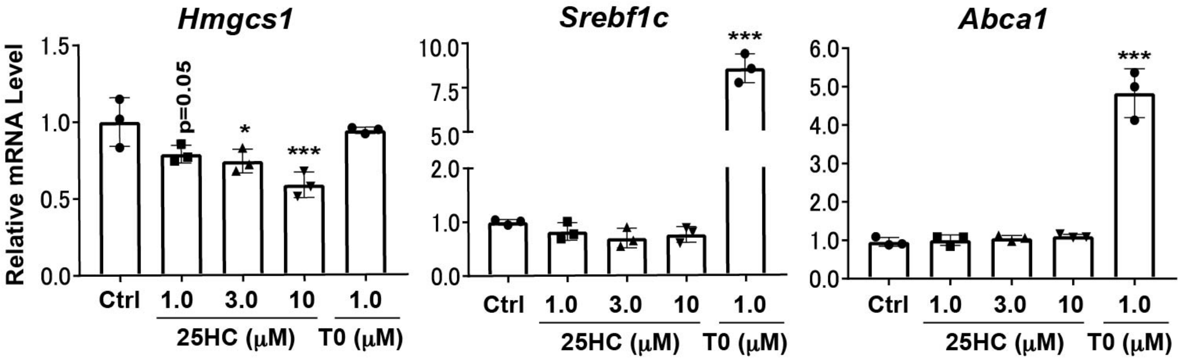

Figure S2. Effects of exogenous 25-HC and T0901317 on SREBP-2 and LXR target gene expression in PRHs (Related to Fig. 1).

PRHs were treated with 25-HC or T0901317 for 6 h at the indicated concentration. mRNA levels of SREBP-2 target genes (*Hmgcs1*) and LXR target genes (*Srebf1c* and *Abca1*) were determined by qPCR. Data represent means ± SD (n = 3). Statistical analyses were performed by one-way ANOVA with Dunnett post hoc test (*p<0.05, **p<0.01, ***p<0.001).

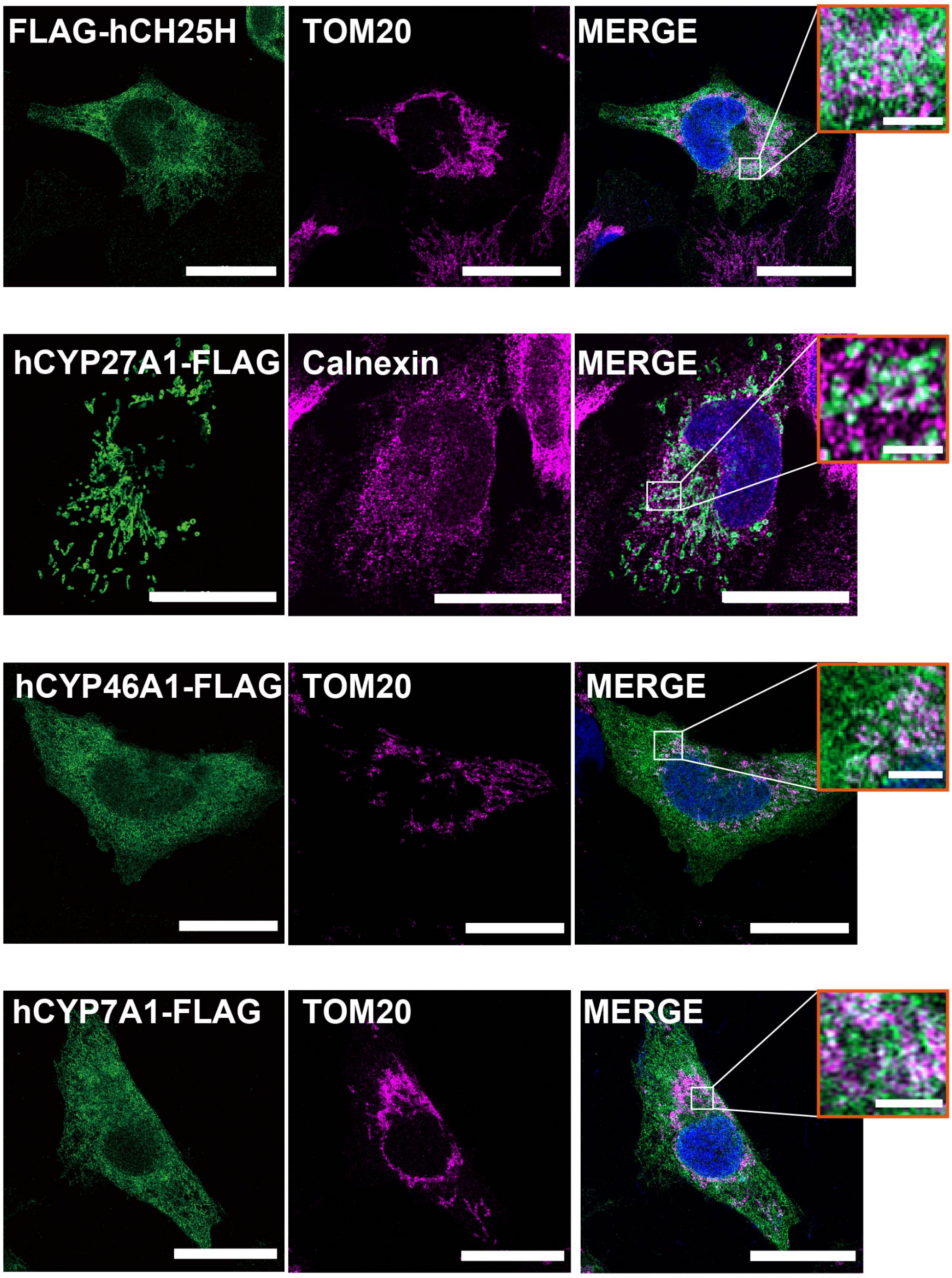

Figure S3. Cellular localization of cholesterol hydroxylases (Related to Fig. 2). A2058 cells transfected with the plasmids (pFLAG-CH25H, pCYP27A1-FLAG, pCYP46A1-FLAG, or pCYP7A1-FLAG) were fixed 2 days after transfection. Cholesterol hydroxylases were labeled with anti-FLAG antibody followed by Alexa 488-conjugated anti-mouse IgG (green). The mitochondria and ER were labeled with anti-Tom20 and anti-calnexin antibodies, respectively, followed by Alexa 568-conjugated anti-rabbit IgG (magenta). Images were taken under a confocal microscopy. Scale bars, main fields, 20 μm; insets, 2 μm.

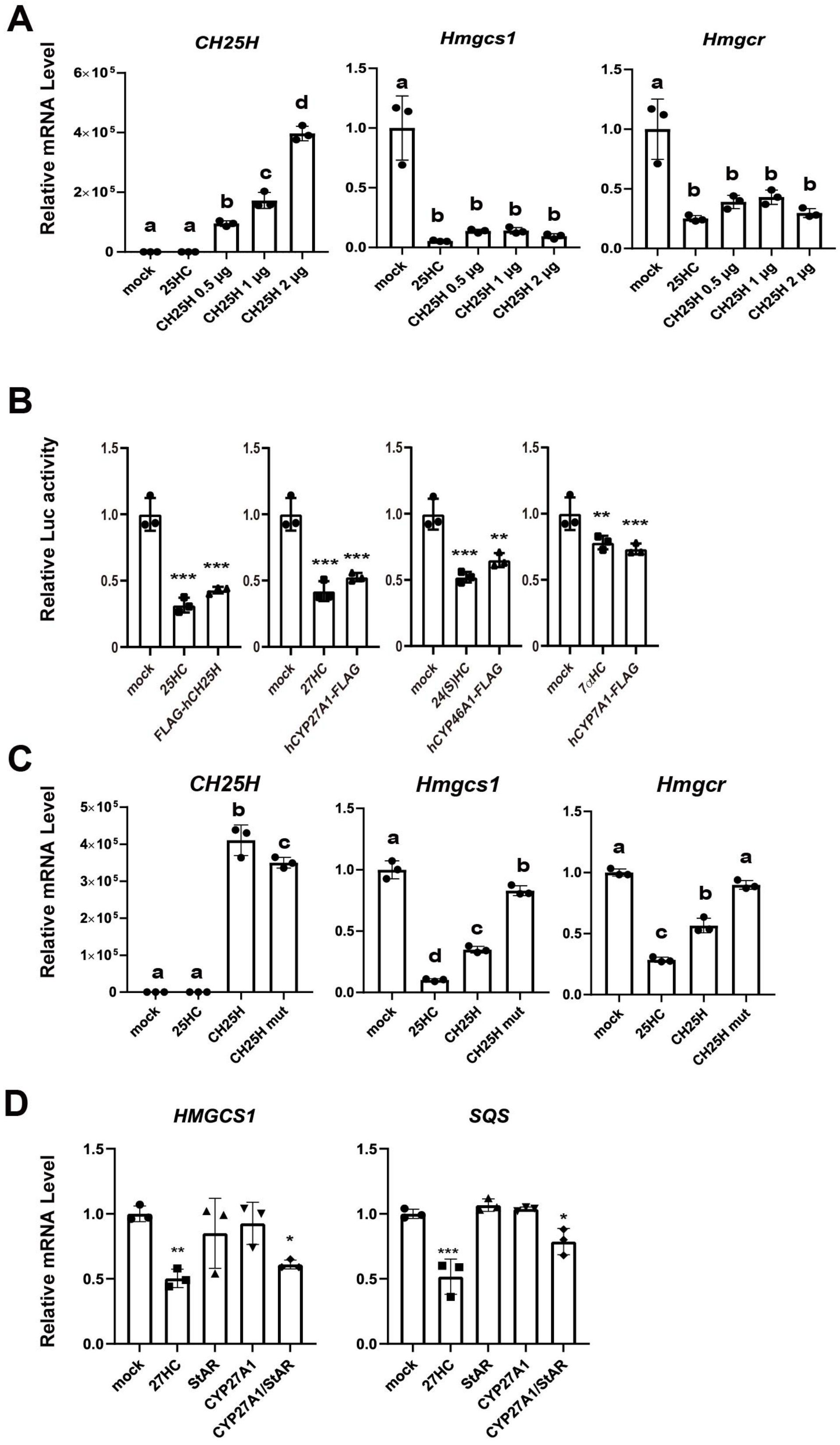

Figure S4. Effects of cholesterol hydroxylase expression on cholesterol homeostatic responses (Related to Fig. 2).

(A) Effects of CH25H expression on SREBP-2 target gene expression. CHO-K1 cells seed into 12-well plates were transfected with pFLAG-CH25H (0.5, 1, or 2 μg/well). Five hours

after transfection, medium was switched to medium containing 0.1% FBS without or with 2.5 μM 25-HC, followed by incubating cells for 16 h. The expression of the indicated genes was analyzed by qPCR.

(B) *Hmgcs1* promoter activity. CHO-K1 cells were transfected with either one of the four hydroxylase expression plasmids (pFLAG-CH25H, pCYP27A1-FLAG, pCYP46A1-FLAG, or pCYP7A1-FLAG) and plasmids for luciferase reporter assay. Mock transfected cells were treated without or with 25-HC, 27-HC, 24S-HC, or 7α-HC (2.5 μM) for 16 h. Luciferase assay was performed as in Experimental procedures.

(C) Effect of mutant CH25H on SREBP-2 target gene expression. CHO-K1 cells seed into 12-well plates were transfected with pFLAG-CH25H (2 μg/well) or pFLAG-CH25H$^{h242Q/H243Q}$ (2 μg/well). Five hours after transfection, medium was switched to medium containing 0.1% FBS without or with 2.5 μM 25-HC, and cells were further incubated for 16 h. The expression of the indicated genes was analyzed by qPCR.

(D) Effect of StAR expression of CYP27A1-dependent repression of SREBP-2 target gene expression. HEK293T cells were transfected with pCYP27A1-FLAG and/or pStAR-FLAG as indicated and incubated in DMEM with 1% FBS in the absence or presence of 27-HC (2.5 μM) for 16 h. mRNA levels of *HMGCS1* and *SQS* were examined by qPCR.

Error bars represent S.D. from three biological replicates. Statistical analyses were performed by one-way ANOVA with Tukey-Kramer (A, C) or Dunnett post hoc test (B, D). Different letters or asterisks (*p<0.05, **p<0.01, ***p<0.001) denote statistical significance.

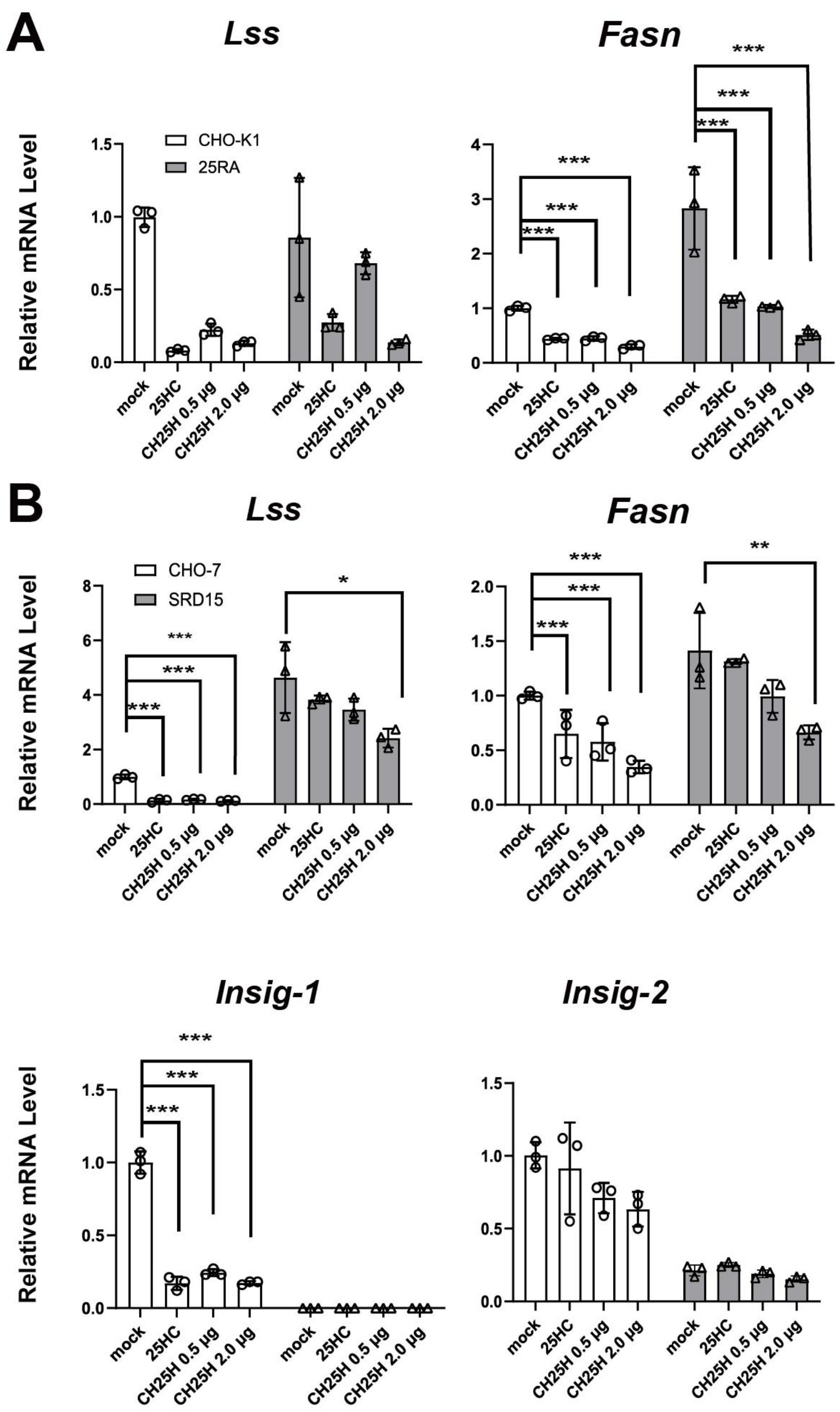

Figure S5. Endogenous 25-HC regulates cellular responses that required Insig1/2 (Related to Fig. 5).

Expression of *Lss*, *Fasn*, *Insig-1*, and *Insig-2* in 25RA (A) and SRD-15 (B) cells. Cells were treated as in Figure 5B and C. mRNA levels were analyzed by qPCR. Data represent means ± S.D. (n = 3). Statistical analyses were performed by one-way ANOVA with Dunnett post hoc test (*p<0.05, **p<0.01, ***p<0.001).

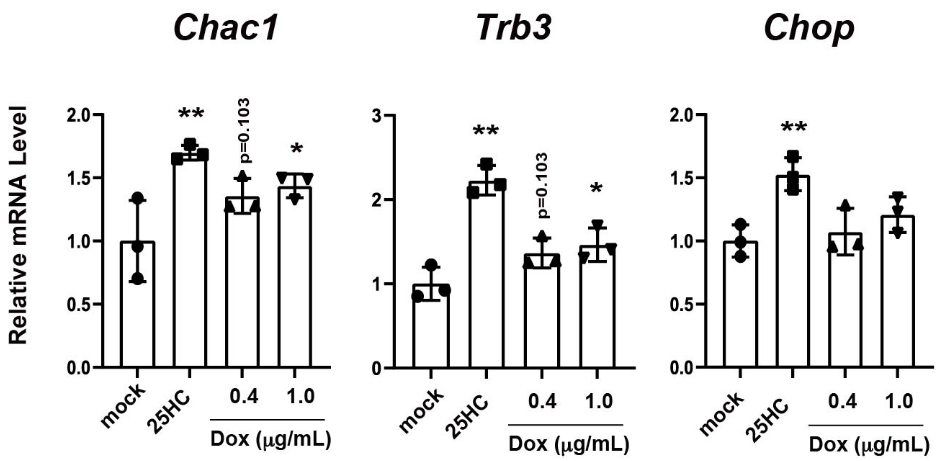

Figure S6. Effects of endogenous 25-HC on the ATF4 axis (Related to Fig. 5).

Effect of CH25H expression on the ATF4 axis. In CHO-hCH25H$^{tet\text{-}on}$ cells, CH25H expression was induced by 0.4 μg/mL or 1 μg/mL Dox as in Figure 3. The expression of ATF4 target genes (*Chac1*, *Trb3*, and *Chop*) was examined by qPCR. Statistical analyses were performed by one-way ANOVA with Dunnett post hoc test (*p<0.05, **p<0.01, ***p<0.001).

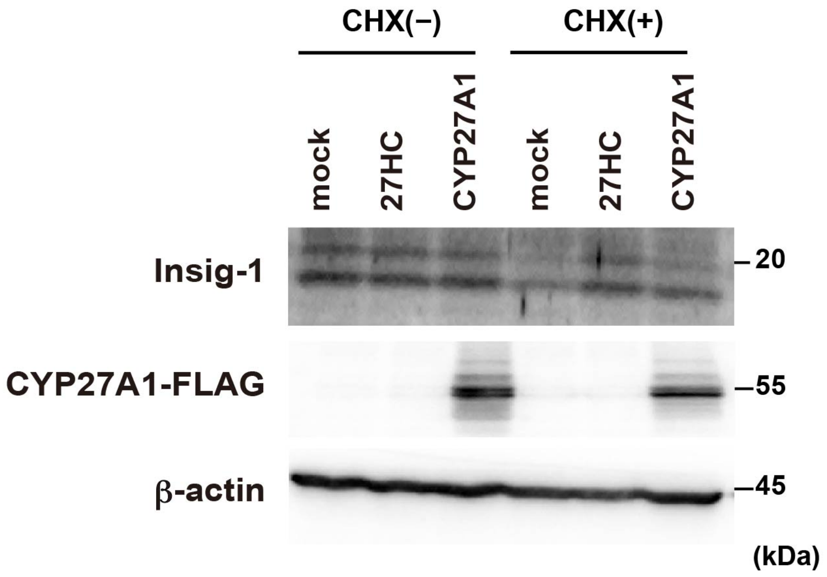

Figure S7. Stabilization of Insig-1 by endogenous 27-HC (Related to Fig. 6).
CHO-K1 cells transfected with pCYP27A1-FLAG or mock vector were treated without or with CHX (50 μM) and 27-HC (2.5 μM) for 2 h as indicated. The expression of Insig-1, CYP27A1-FLAG, β-actin was assessed by immunoblotting.
